# Pests, diseases, and aridity have shaped the genome of *Corymbia citriodora*

**DOI:** 10.1038/s42003-021-02009-0

**Published:** 2021-05-10

**Authors:** Adam L. Healey, Mervyn Shepherd, Graham J. King, Jakob B. Butler, Jules S. Freeman, David J. Lee, Brad M. Potts, Orzenil B. Silva-Junior, Abdul Baten, Jerry Jenkins, Shengqiang Shu, John T. Lovell, Avinash Sreedasyam, Jane Grimwood, Agnelo Furtado, Dario Grattapaglia, Kerrie W. Barry, Hope Hundley, Blake A. Simmons, Jeremy Schmutz, René E. Vaillancourt, Robert J. Henry

**Affiliations:** 1 HudsonAlpha Institute for Biotechnology, Huntsville, AL USA; 2 University of Queensland/QAAFI, Brisbane, QLD Australia; 3 Southern Cross Plant Science, Southern Cross University, Lismore, NSW Australia; 4 School of Natural Sciences, University of Tasmania, Hobart, TAS Australia; 5 ARC Training Centre for Forest Value, University of Tasmania, Hobart, TAS Australia; 6 Scion, Rotorua, New Zealand; 7 Forest Industries Research Centre, University of the Sunshine Coast, Sippy Downs, QLD Australia; 8 EMBRAPA Genetic Resources and Biotechnology, Brasília, Brazil; 9 Institute of Precision Medicine & Bioinformatics, Camperdown, NSW Australia; 10 Department of Energy Joint Genome Institute, Berkeley, CA USA; 11 Genomic Science Program, Universidade Catolica de Brasilia, Taguatinga, Brazil; 12 Joint BioEnergy Institute, Emeryville, CA USA

**Keywords:** Plant evolution, Forestry

## Abstract

*Corymbia citriodora* is a member of the predominantly Southern Hemisphere Myrtaceae family, which includes the eucalypts (*Eucalyptus*, *Corymbia* and *Angophora*; ~800 species). *Corymbia* is grown for timber, pulp and paper, and essential oils in Australia, South Africa, Asia, and Brazil, maintaining a high-growth rate under marginal conditions due to drought, poor-quality soil, and biotic stresses. To dissect the genetic basis of these desirable traits, we sequenced and assembled the 408 Mb genome of *Corymbia citriodora*, anchored into eleven chromosomes. Comparative analysis with *Eucalyptus grandis* reveals high synteny, although the two diverged approximately 60 million years ago and have different genome sizes (408 vs 641 Mb), with few large intra-chromosomal rearrangements. *C. citriodora* shares an ancient whole-genome duplication event with *E. grandis* but has undergone tandem gene family expansions related to terpene biosynthesis, innate pathogen resistance, and leaf wax formation, enabling their successful adaptation to biotic/abiotic stresses and arid conditions of the Australian continent.

## Abstract (web summary)

Healey and colleagues presented a reference sequence assembly of *Corymbia citriodora* (spotted gum), a tree which is crucial for timber, pulp, and paper, as well as carbon sequestration and essential oil production.

## Introduction

Embedded within genomes are the footprints of climatic and evolutionary history in which progenitor lineages have undergone selection^1^. The detection of these footprints can provide insight into the historic conditions experienced by organisms of interest, with plant genomes in particular often exhibiting distinct adaptive signatures due to their sessile nature^2^. Forest trees, as some of the longest-lived plants and therefore exhibiting strong local adaptation, are important for the renewable delivery of materials and energy worldwide, play a key role in carbon cycling and storage, and affect rainfall patterns^3^. Angiosperms (flowering plants) abound with tree species that occur in most taxonomic orders^4^. However, our current insights into the evolution of their genomes are primarily based on comparative analysis of Northern Hemisphere deciduous taxa, within families such as the Rosaceae^5–7^, Salicaceae^8^, Fagaceae^9,10^, and Oleaceae^11,12^. Although *Eucalyptus grandis* was the second forest tree genome to be assembled^13^, there has been little progress in unravelling key aspects of genome organization and evolution within the predominantly Southern Hemisphere family Myrtaceae to which it belongs. The Myrtaceae is a diverse, ecologically and economically important plant lineage (~5,700 species; 132 genera^14^) that includes tree species such as clove (*Syzygium*), guava (*Psidium*), tea-trees (*Melaleuca* and *Leptospermum*) and mangroves (*Osbornia*)^15^. It also includes the globally grown eucalypts, which are endemic to Australia and islands to its north^16^.

Eucalypts comprise over 800 species, belonging to three closely related genera—*Angophora*, *Corymbia,* and *Eucalyptus*^17^. Eucalypts diverged from their closest Myrtaceae relative, Syncapieae, approximately 65–68 million years ago (MYA)^18^ and radiated into diverse environments undergoing rapid expansion immediately after the Cretaceous, followed by domination during the Paleocene-Eocene thermal maximum (~55 MYA) and climate aridification in the mid-late Miocene (~15 MYA)^19–21^. *Eucalyptus*, the largest genus, diverged from the *Angophora-Corymbia* lineage ~60 MYA, which roughly corresponds to the separation of the Australian continent from Antarctica [83–45 MYA], at which time the two had long separated from other Gondwana land masses^16,22^. *Eucalyptus* is widely distributed across the Australian continent but is largely consolidated in the more southern bioregions^23^. In contrast, *Corymbia* and *Angophora* are largely absent from most southern forests, having radiated through coastal and sub-coastal regions of eastern Australia, with *Corymbia* also extending across the northern, tropical ‘top-end’ of Australia^24^. These differences in the geographic range likely reflect evolutionary, adaptive differences between the *Angophora*-*Corymbia* and *Eucalyptus* lineages. Climate niche adaptation is signaled by field trials showing *Corymbia* species are typically more cold-averse and drought tolerant than *Eucalyptus*^25,26^ and thrive in a wide range of rainfall conditions (0.6–2.0 m/year)^27^ and marginal soils^28^. In terms of biotic environment, insects and fungal diseases have represented the primary pest challenge for both lineages, with genus-level differences in susceptibility often evident^29,30^. The emblematic defensive strategy taken within the Myrtaceae has been the generation of a diverse range of terpenoids^31^, with complex profiles matching their diversity and a corresponding expansion of the terpene synthase gene family^32,33^.

Here we present the genome assembly of *C. citriodora* subsp. *variegata* (CCV), a new Myrtaceae reference sequence for a taxa important for timber, pulp and paper, carbon sequestration, and essential oil production in areas considered too marginal for other productive species due to pests, diseases, and drought. Despite their divergence and adaptive radiation across different biomes in Australia, the genome structure among *Eucalyptus grandis* and CCV is highly conserved (2*n* = 22). *Corymbia* has retained evidence of an ancient (109 MYA) Myrtales whole-genome duplication (WGD) event and exhibits post-divergence gene family expansions related to terpene synthesis and biotic/abiotic stress resistance. This new genome sequence will enable comparative genomic studies for the dominant hardwood taxa in the Southern Hemisphere and will serve as a valuable resource for further development of this strategic woody biomass resource for manufacturing and bioenergy sectors.

## Results

### Genome assembly and annotation

*Corymbia citriodora* subsp. *variegata* genotype CCV2-018 was selected for reference sequencing due to its wide use as a parent in the spotted gum breeding program of the Queensland Department of Agriculture and Fisheries, and its use for the generation of interspecific hybrids for investigating pulp and bioenergy production^34^. In brief, 129 Gb of raw data was generated from two Illumina HiSeq2500 libraries (2 × 150 bp paired end; insert sizes: 400 and 800 bp), representing ~320× sequencing coverage of the genome. The genome assembly was generated using a modified version of Arachne (v.20071016)^35^. Contig assembly and initial scaffolding steps produced 37,263 contigs in 32,740 scaffolds (N50 length: 132.6 Kb), totaling 563.0 Mb. Gap patching on the scaffolds was performed using ~25× PacBio reads (N50 length: 17,094 bp) and QUIVER (www.github.com/PacificBiosciences/GenomicConsensus). Final scaffolding was completed using SSPACE-Standard^36^ (Version 2.0) with Nextera long mate pair libraries (insert size 4 Kb and 8 Kb), resulting in a 537.9 Mb assembly (16,786 scaffolds; 20,979 contigs) with a scaffold N50 of 312 Kb.

**Fig. 1:**
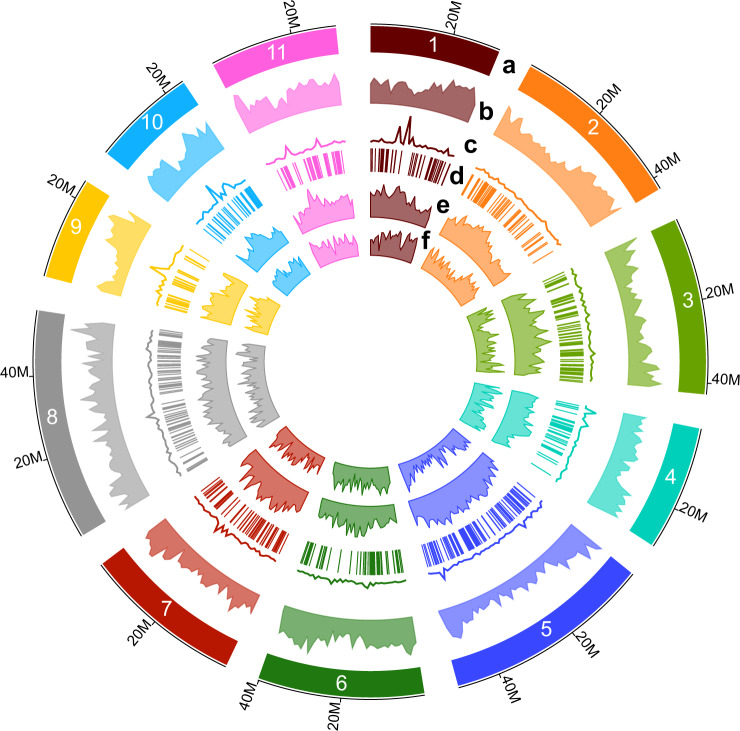
Main genome features of *Corymbia citriodora* subspecies *variegata* (CCV). **a** The eleven chromosomes of CCV. Numbers along the outside track denote chromosome length in megabases. **b** Gene density (gene number per megabase; 5–143). **c** Average expression (rpkm; 0.5–150) among collected tissues for RNASeq per megabase. **d** Tandem gene arrays. **e** Percent repetitive content per megabase (5–61%). **f** Number of heterozygous SNPs per megabase (230–7,282).

To anchor the scaffolds into chromosomes, the sequences were ordered and oriented into 11 pseudomolecules (Fig. 1; Supplementary Data File 1) using *Corymbia* genetic maps^37^. Three high-density linkage maps were generated from two *C. torelliana* × *C. citriodora* subsp. *variegata* hybrid crosses (CT2-050 × CCV2-054, CT2-018 × CCV2-054) genotyped with Diversity Arrays DArTseq technology^38^, and contigs were anchored to the marker sequences using ALLMAPS^39^ (Supplementary Fig. 1). The average Spearman correlation coefficient of centimorgan (cM) positions for genetic map markers from all three linkage maps and physical locations on scaffolds was 0.96. The pseudomolecules range in size from 24.8 Mb (Chromosome 9) to 55.7 Mb (Chromosome 8). The total genome size of chromosome anchored scaffolds (*n* = 4,033) was 412 Mb (408 Mb in contigs), which is close to the estimated genome size of 370–390 Mb, based on flow cytometry^40^. Global genome heterozygosity was estimated at ~0.5% through calling heterozygous SNPs against repeat-masked bases in chromosomes. The evaluation of the protein-coding annotation completeness with single-copy orthologs on the assembly was undertaken using Benchmarking Universal Single-Copy Orthologs (BUSCO; Supplementary Table 1)^41^, receiving a 95.1% score, suggesting high quality and completeness. In addition, 90% of derived single-copy genes in *E. grandis* were also single copy on CCV chromosomes, suggesting that pseudomolecule construction was complete and alternative haplotypes had not been introduced into the main genome assembly (Supplementary Fig. 2).

To annotate the genome, RNA was collected from five separate tissues (expanded leaves [EL], unexpanded leaves [UL], flower buds [FB], flower initials [FI], photosynthetic bark cortex [BA]) (Supplementary Figs. 3, 4) and was used for de novo gene model prediction. The final annotation of protein-coding gene products comprised 35,632 primary transcripts and 10,019 alternative transcripts for a total of 45,651 transcript models. The set of primary transcripts had a mean length of 3.4 Kb, a mean of 4.8 exons, with a median exon length of 176 bp and a median intron length of 202 bp. The total amount of repetitive content captured in the pseudomolecules was 146.5 Mb, which represents ~35.8% of the genome (Table 1). The repetitive content was primarily comprised of Class I Retro transposable elements (19.48%) and Class II DNA transposable elements (5.42%).

**Table 1:** Assembly and genome statistics for *Corymbia citriodora* subspecies *variegata* (v2.1- Phytozome v13) and *Eucalyptus grandis* (v2.0- Phytozome v13).

	*C. citriodora* ssp. *variegata*	*E. grandis*
Bases in chromosomes (Megabases [Mb])	408	641
Number of scaffolds in chromosomes	4,033	4,952
Number of chromosomes	11	11
Contig N50 length	185.5 Kb	67.2 Kb
Scaffold N50 length	31.5 Mb	57.5 Mb
GC content	39.1%	39.3%
Repetitive content	35.78%	43.96%
Retro transposable elements (RNA; Class I)	19.48%	22.06%
DNA transposable elements (Class II)	5.42%	7.22%
Total number of primary gene models	35,632	36,349
Total number of transcript models	45,651	46,280

### Comparative genome analysis

**Fig. 2:**
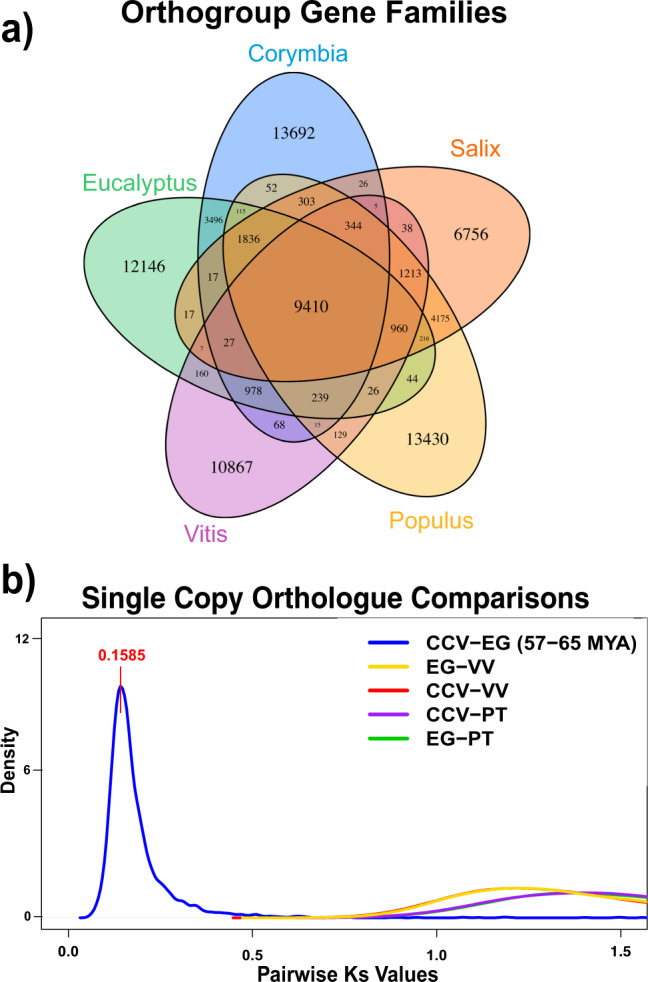
Orthologous gene groups among woody plant genomes. **a** Shared orthogroups among *Corymbia citriodora* ssp. *variegata*, *Eucalyptus grandis*, *Populus trichocarpa*, *Salix purpurea,* and *Vitis vinifera*. **b** Single-copy ortholog synonymous substitution rate (Ks) comparisons to date the *Corymbia*/*Eucalyptus* divergence. Blue line- Synonymous substitution rates among CCV and EG orthologs. Yellow line- Synonymous substitution rates among EG and VV orthologs. Red line- Synonymous substitution rates among CCV and VV orthologs. Purple line- Synonymous substitution rates among CCV and PT orthologs. Green line- Synonymous substitution rates among EG and PT orthologs. PT *Populus trichocarpa*, CCV *Corymbia citriodora* subsp. *variegata*, EG *Eucalyptus grandis*, VV *Vitis vinifera*.

Divergence between *Corymbia* and other woody angiosperm genomes^42^ (*Eucalyptus grandis*^13^, *Salix purpurpea* [willow]^43^, *Populus trichocarpa* [poplar]^8^, *Vitis vinifera* [grape]^44^) was investigated using the synonymous mutation rate (Ks) among single-copy orthologous genes. All-on-all Diamond alignment hits were filtered based on syntenic blocks (as a complimentary measure to orthology), finding 9,410 syntenic orthogroups among the five genomes and 3,496 eucalypt-specific (shared among *E. grandis* and CCV) gene families (Fig. 2a; Supplementary Data File 2). Among the syntenic orthogroups, 2,942 contained single-copy orthologs and were used to estimate the synonymous substitution rate (Ks) within *Corymbia*. Based on the median Ks peak among CCV and *E. grandis* (0.1585) (Fig. 2b; Supplementary Data File 3) and estimated divergence times based on the fossil record (57.2, 58.5, 64.6 MYA)^16^, the synonymous mutation rate (site/year) among orthologs was estimated between 1.385 × 10^−9^ and 1.227 × 10^−9^.

While these mutation rates are consistent with those observed within Salicaceae^45^, they are substantially slower (3.8–4.0 fold) than SNP- based population estimates from *E. grandis* (4.93 × 10^−9^)^46^. To investigate this result further, we calculated estimates of the population mutation rate parameter for *Corymbia* from re-sequencing data of the parental genotype (CCV2-018), as well as four unrelated CCV genotypes (CCV2-019, CCV2-025, CCV2-045, CCV2-046). The measures of population mutation rate (4N_e_μ) obtained from the genotypes following maximum likelihood estimators based on the shotgun sequence data ranged between 7.12 × 10^−3^ and 8.32 × 10^−3^ (average 7.86 × 10^−3^). For the purpose of the comparison with *E. grandis*, we assumed an ancestral population size of 112,421, which is consistent with the past demographic history of that species^46^. On this basis, the mutation rate per site per generation in CCV is estimated between 1.59 × 10^−8^ and 1.85 × 10^−8^, which is consistent with the Ks mutation result if a generation time of about 15 years is assumed for CCV (1.85 × 10^−8^ /15 y = 1.23 × 10^−9^ site/year). However, it is worth noting that the true generation time of CCV is unknown, as CCV undergoes mass flowering^47^, and (unlike *E. grandis*) forms lignotubers through which they can regenerate^48^.

Assuming the above assumptions are plausible, the nucleotide diversity seems to be lower in CCV than in *E. grandis*, while the overall chromosome recombination rates appear to be consistent between both species (CCV = 2.85 cM/Mb; *E. grandis* = 2.98 cM/Mb)^49^ (Supplementary Table 2). Other than recombination, one factor that negatively correlates with diversity in genomes is the density of targets for purifying selection, which has been often approximated by the density of coding sequence^50^. In a scenario in which long-term effective population size and recombination remain equal between the species, the higher density of coding sequences in CCV due to its smaller genome could be a contributing factor for the reduction of its diversity. It might be important to carry out a further detailed investigation if the apparent reduction of diversity in CCV is predominantly an effect of the increase in density of targets for selection due to changes in chromosome size. An alternative explanation may relate to differences in the ancestral population sizes or its patterns of variation in comparison to *E. grandis* demographics.

**Fig. 3:**
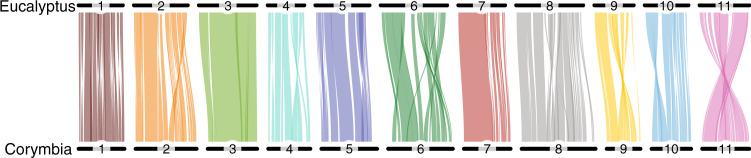
Intra-chromosomal syntenic blocks among *Eucalyptus grandis* and *Corymbia citriodora* subsp *variegata*. Numbers represent individual chromosomes. Minimum number of genes per block is 25.

Despite differences in their genome size and ~60 MYA divergence, CCV and *E. grandis* have few large-scale intra-chromosomal rearrangements and have retained large syntenic blocks (Fig. 3; Supplementary Data File 4). Both species have 11 chromosomes (2*n* = 22), with chromosomes 1, 3, 5, and 7 being largely 1:1 syntenic. Chromosomes 4, 8, 9, 10, and 11 contain major inversions, and chromosomes 2 and 6 harbor inverted intra-chromosomal translocations. These major chromosomal re-arrangements have been previously described^37^, but chromosome 11 in the genome assembly was inverted relative to the genetic map to maximize synteny with *E. grandis*. Within Myrtaceae, a chromosome number of 11 (2*n* = 22) has been widely conserved across most major clades, with some exceptions of polyploidy (2*n* = 33,44,66 [3x,4x,6x]) occurring within *Leptospermum*, *Psidium*, and *Eugenia*^15^. Globally, 71% (*n* = 25,357) of CCV genes were retained in large intra-chromosomal syntenic blocks with *E. grandis* and 86% average identity between protein sequences (top hit among CCV and *E. grandis* primary proteins). An average of 17 syntenic blocks were detected on each chromosome. Chromosome 3 was the most syntenic with 98% of genes captured in six blocks and the largest block containing 89% of all chromosome 3 genes (Table 2; Fig. 3; Supplementary Data File 4). Chromosome 6, despite multiple inverted translocations, maintained 75.3% of genes in 19 syntenic blocks. The overall correlation (*r*) between CCV and *E. grandis* chromosome sizes is 0.88 (*n* = 11; *p* = 0.003).

**Fig. 4:**
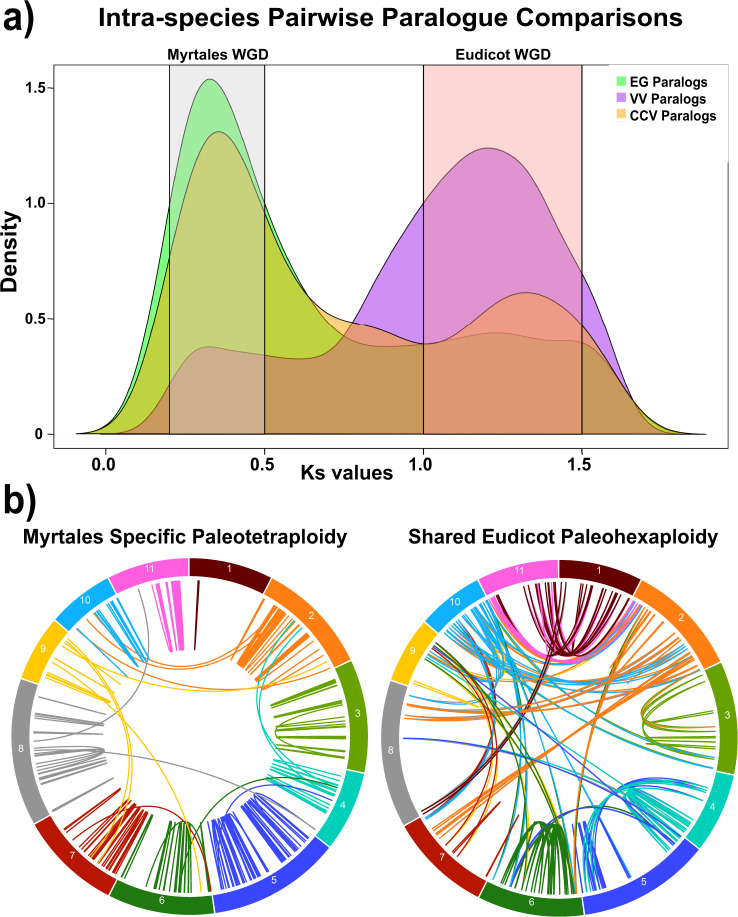
Patterns of paralog retention in *Corymbia* resulting from whole-genome duplications (WGD). **a** Intra-species retained paralogous sequences from the shared eudicot and Myrtales specific WGD events. Ks- synonymous mutation rate. Green distribution- Ks values among paralogous sequences within *E. grandis*. Yellow distribution- Ks values among paralogous sequences within *C. c.variegata*. Purple distribution- Ks values among paralogous sequences within *Vitis vinifera*. Gray box- paralogous sequences derived from the Myrtales WGD event. Pink Box- paralogous sequences derived from the Eudicot WGD event. CCV *Corymbia citriodora* subsp. *variegata*, EG *Eucalyptus grandis*, VV*Vitis vinifera*. **b** *Corymbia* chromosomal dispersal pattern of paralogs from the eudicot and Myrtales WGD event. Lines between chromosomes represent intra-specific paralogous sequences that arose from each duplication.

**Table 2:** Comparison of chromosome gene content and synteny between *Corymbia citriodora* subspecies *variegata* and *Eucalyptus grandis*.

Chromosome	Total # of genes on Chr	Genes in syntenic blocks	Percent genes in syntenic blocks	Total number of syntenic blocks	Number of genes in largest syntenic block	Percent of genes captured by largest block
1	2,436	2,235	91.7	25	426	17.5
2	3,246	2,810	86.6	23	677	20.9
3	2,618	2,574	98.3	6	2,297	87.7
4	2,234	1,684	75.4	11	403	18.0
5	2,958	2,373	80.2	13	746	25.2
6	3,427	2,582	75.3	19	906	26.4
7	2,478	2,206	89.0	15	1,523	61.5
8	3,923	3,298	84.1	29	600	15.3
9	1,792	1,463	81.6	13	422	23.5
10	2,099	1,874	89.3	19	335	16.0
11	2,497	2,258	90.4	17	710	28.4

Despite high synteny, Myrtaceae genomes vary considerably in size^13,15^. Across the entire Myrtaceae family, the 1n genome size range of diploids (based on flow cytometry) is approximately fivefold, from 234 Mb (*Myrciaria glazioviana*; pg/1C = 0.239) to 710 Mb (*Eucalyptus saligna*; largest eucalypt; pg/1C = 0.735) and 1.1 Gb (*Melaleuca leucadendra*; pg/1C = 1.100)^15,51,52^. The difference in genome size between CCV (408 Mb) and *E. grandis* (641 Mb) was 233 Mb, of which ~139 Mb could be attributed to repetitive content (35.8% vs 43.9%, respectively when compared using the same pipeline). While this observation is consistent with other plant genomes where repeat content contributes to genome size differences^53^, comparisons between *E. grandis* and *E. globulus* (section *Symphyomyrtus;* ~36 MYA divergence)^13^ showed a genome size difference of 111 Mb (i.e., 641 Mb and 530 Mb respectively), the majority of which was attributable to non-repetitive *E. grandis* specific sequences distributed throughout the genome (88.7 Mb). *Eucalyptus grandis* compared to the more distantly related, recently sequenced draft assembly of *E. pauciflora* (subgenus *Eucalyptus*)^53^ found that while the genome of *E. pauciflora* (594 Mb) was 16% smaller than *E. grandis*, its repeat content was greater (44.77% versus 41.22%).

### Whole-genome duplications

Following the eudicot gamma WGD event (~140 MYA-paleohexaploidy)^54^, eucalypts underwent a lineage-specific paleotetraploidy event (~109 MYA), which coincides with the Myrtales divergence from other Rosids^13,55^ and is considerably older than other WGD events that have occurred in poplar, *Arabidopsis,* and soybean^45,56,57^. Evidence of this event is present in *Corymbia*, based on Ks values of syntenic paralogous sequences. Ks values among intra-specific paralogs of CCV, *E. grandis* and *V*. *vinifera* revealed a clear signal of the shared eudicot paleohexaploidy event^54^ (Ks ~1.2) and the Myrtales specific paleotetraploidy event (Ks ~0.4) (Fig. 4a; Supplementary Data File 5). Illustration of these paralogous pairs shows a similar intra-chromosomal dispersion pattern within CCV (Fig. 4b; Supplementary Data File 5) as *E. grandis* (Myburg et al.^13^: Fig. 2b). KEGG pathway enrichments among *Corymbia* paralogs within the Myrtales WGD peak (*n* = 528) showed significant enrichment for the biosynthesis of unsaturated fatty acids (*P* = 0.02), nitrogen metabolism (*P* = 0.03), plant-pathogen interaction (*P* = 0.03), and sesquiterpenoid and triterpenoid biosynthesis (*P* = 0.02) (Supplementary Data File 5; Supplementary Data File 6). The importance and prevalence of terpene biosynthesis is well documented in the eucalypts as a mechanism for mediation of abiotic/biotic stresses^33^. Unsaturated fatty acids are a key component in the waxy leaf cuticle of *Eucalyptus*, which not only protect against temperature stress through osmo-regulation of water, but are also implicated in resistance to fungal pathogens such as Myrtle rust (*Austropuccinia psidii*)^58^, which threatens 1,285 species of Myrtaceae^59^.

*Eucalyptus grandis* chromosome 3 has previously been identified as the most conserved, maintaining synteny with *P. trichocarpa* chromosome XVIII despite being more than 100 million years diverged^13^. CCV chromosome 3 also maintains this pattern, while other conserved syntenic blocks among CCV and *E. grandis* were dispersed among multiple *P. trichocarpa* chromosomes (e.g., CCV chromosome 5; Supplementary Data File 6). Myburg et al.^13^ postulated that chromosome 3 (and chromosome XVIII in *P. trichocarpa*) each represent a single copy of the ancestral eudicot chromosome A4. After the Myrtales-specific WGD, chromosome 3 homologs fused, as evidenced by all retention of paralogs from the WGD residing on chromosome 3; a pattern shared by CCV (Fig. 4b; Supplementary Data File 5). Myburg et al.^13^ proposed that the maintenance of synteny between *E. grandis* and *P. trichocarpa* was possibly due to either: (1) favored preservation of perennial woody-habit genes, or (2) introduced internal centromeres and telomeres (3 Mb and 74 Mb within *E. grandis*) that have repressed subsequent recombination and gene expression^13^. To investigate this possibility, differences in gene expression, recombination, and synteny on CCV chromosome 3 were considered. We observed no significant differences (ANOVA; *P* > 0.05) in the average gene expression within chromosome 3 and other chromosomes (Supplementary Fig. 7), nor higher repetitive content. Looking broadly across all chromosomes, chromosome 3 had lower recombination than average (2.67 cM/Mb; mean: 2.85 cM/Mb), but not the lowest rate (chromosome 5: 2.39 cM/Mb; Supplementary Table 2). The mean recombination rate of CCV is consistent with *E. grandis, E. globulus, and E.urophylla*^49^, but it is worth noting that within *E. globulus*, recombination rates for chromosomes 3 and 5 were significantly less than other chromosomes, likely due to their large size. CCV chromosomes are more uniform in length than *E. grandis* (CCV average chromosome length = 37.5 Mb; standard deviation = 10 Mb) with similar differences in recombination rate and number of crossover events (Supplementary Tables 2 and 3).

To investigate the possibility that conserved woody-habit genes may require retention of syntenic order, all CCV chromosome 3 orthologs that are maintained within syntenic blocks in both *E. grandis* and *P. trichocarpa* (chromosomes XVIII and VI [which share synteny due to the Salicoid WGD event]) were extracted (1:1:2; *n* = 173 genes) (Supplementary Fig. 8; Supplementary Data File 7) and compared for enriched gene functions. The top KEGG enrichment pathways for this gene set included phenylalanine and tyrosine metabolism (*P* = 0.001; *P* = 0.003; respectively), alkaloid biosynthesis (tropane and isoquinoline) (*P* = 0.02), and glycerolipid metabolism (*P* = 0.02), each of which play critical roles in plant primary and specialized metabolism (e.g., monolignol biosynthesis), cell wall formation, defense and stress signaling^60–63^. Regarding plant defense, quantitative trait loci (QTLs) for fungal disease resistance have been repeatedly reported on chromosome 3 in *E. grandis* from several independent studies using different methods of genetic evaluation in breeding populations, clearly demonstrating a major involvement of this chromosome in the pathogen resistance response^64^. While these results favor the syntenic maintenance of critical gene functions on chromosome 3, gene order conservation needs to be examined further across a larger cohort of woody angiosperm genomes.

### *Corymbia* gene family analysis

Comparative genomics between CCV and *E. grandis* allowed investigation of gene family expansions that had occurred since eucalypts diverged from other Rosids, as well as expansions specific to CCV itself. Within our constrained syntenic orthogroups, 124 had eucalypt-specific expansions (orthogroups with >5 genes and >70% of genes derived from both CCV and *E. grandis*) containing 1,494 genes (735-CCV; 759-*E. grandis*). Functional KEGG pathway enrichments within these expansions included the molecular processes of phenylpropanoid biosynthesis (*P* = 0.00006), cyanoamino acid metabolism (*P* = 0.006), pentose/glucuronate interconversions (*P* = 0.01), isoflavonoid biosynthesis (*P* = 0.02), monoterpenoid biosynthesis (*P* = 0.03), glucosinolate biosynthesis (*P* = 0.03), and plant-pathogen interaction (*P* = 0.04) (Supplementary Table 4). These expansions are consistent with general response mechanisms for biotic/abiotic stress, where carbohydrate pathway activation enables rapid signaling and energy for terpene and secondary metabolite biosynthesis^65–68^.

Similarly, we characterized gene families that had undergone species-specific expansions within *E. grandis* and CCV relative to the other woody angiosperm genomes. Within *E. grandis*, investigation of expanded gene families (orthogroups with at least five genes and ≥50% derived from *E. grandis*) found 179 expanded orthogroups containing 1,479 genes. KEGG pathway enrichment analysis within this dataset found the greatest enrichments for galactose metabolism (*P* = 0.00008), phenylpropanoid biosynthesis (*P* = 0.006), flavonoid biosynthesis (*P* = 0.002), pentose and glucuronate interconversions (*P* = 0.002), and alpha-linolenic acid metabolism (*P* = 0.003) (Supplementary Table 5). Within CCV (using the same criteria for expansion noted above), there were 75 expanded gene family orthogroups containing 501 genes. Functional enrichments within these expanded gene families found significant KEGG pathway enrichments relating to plant-pathogen interaction (*P* = 0.003), phenylpropanoid biosynthesis (*P* = 0.003), ether lipid metabolism (*P* = 0.02), sesquiterpenoid and triterpenoid biosynthesis (*P* = 0.02), and cutin, suberine and wax biosynthesis (*P* = 0.03) (Supplementary Table 6). Considering the substantial overlap among significant KEGG enrichments between CCV and *E. grandis* expansions, genes associated with these terms were mapped to their closest ortholog among shared KEGG pathways (Supplementary Figs. 9–12). We found that CCV and *E. grandis* both had both similar (e.g., cutin and sesquiterpenoid biosynthesis) and separate expansions (e.g., phenylpropanoid biosynthesis).

Specifically, when comparing overlaps among species-specific expanded gene families within plant-pathogen interaction KEGG pathways, CCV displayed signatures of expansion in gene families that were absent in *E. grandis* (Supplementary Fig. 12). Gene families with shared expansions in both CCV and *E. grandis* were related to disease resistance protein 2 (*rps2*) and mitogen-activated protein kinase kinase kinase 1 (*mekk1*), which are part of separate stress response pathways within the cytoplasm. *Mekk1* is a mitogen-activated protein kinase (MAPK) signal cascading gene within the pathogen-associated molecular pattern triggered immunity (PTI) pathway that is tightly associated with abiotic stress response such as temperature, drought, salinity, as well as wounding^69^. *Rps2* is a resistance NB-LRR gene that upon recognition with bacterial effector proteins, generates an effector-triggered immune (ETI) response and can elicit a localized hypersensitive response (HR) where cells undergo programmed death to prevent pathogen spread^70^. Investigation of CCV-specific gene family expansion revealed separate expansions in the same pathogen interaction pathways: *rpm1*, *fls2,* and *bak1*/*bkk1*. Similar to *rps2*, *rpm1* is an R gene and elicits an ETI/HR response upon detection of bacterial effector proteins^71,72^. *Fls2* and *bak1* however, are part of the PTI immune response pathway, both being plasma-membrane bound receptor kinases that form a signaling complex, that upon activation, cascade signals to cytoplasmic kinases as part of PTI responses. *Fls2* recognizes a specific peptide sequence of bacterial flagellin, and while *bak1* is part of the same plasma membrane complex, it can initiate signaling independently of *fls2*, recognizing the EF-Tu bacterial receptors, lipopolysaccharides, peptidoglycans and whose function is critical as part of the plant innate immune response^73,74^.

Based on the prevalence of tandem gene arrays in *E. grandis*, we investigated tandem gene duplication in CCV as a mechanism for gene family expansion whose functions are enriched for climate niche adaptation for hot season rainfall and semi-arid environments^75^. CCV has a large number of tandemly duplicated genes, with a similar number of arrays in extended syntenic blocks as *E. grandis* (8,366 vs 8,679; 23% vs 24% of all genes, respectively). This number is lower than previously reported for *E. grandis* (*n* = 12,570)^13^, as we used MCScanX^76^ as our standardized, more conservative, methodology for identifying tandem repeats within each genome, as these can often be difficult to define. As hypothesized, there was an 81% overlap (*n* = 408) between CCV-specific gene family expansions and tandem duplicates which were significantly enriched for the same KEGG pathways, thus tandem gene duplication appears to be a major mechanism of gene family expansion in eucalypts.

**Fig. 5:**
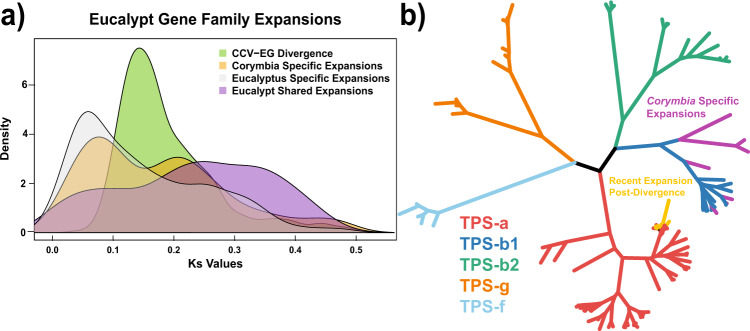
Relative timing of eucalypt gene family expansions. **a** Synonymous substitution rates (Ks) among orthologs derived from shared eucalypt expansion and those that are *E. grandis* and *C. c. variegata* specific. Green distribution- *Corymbia*-*Eucalyptus* divergence peak among orthologs. Gray distribution- *E. grandis* expanded orthogroups. Orange distribution- *C. c. variegata* expanded orthogroups. Purple distribution- *E. grandis* and *C. c. variegata* shared expanded orthogroups. **b** *C. c. variegata* terpene gene families. Red branches- Terpene synthase family-a genes. Blue branches- Terpene synthase family-b1 genes. Green branches- Terpene synthase family-b2 genes. Orange branches- Terpene synthase family-g genes. Light-blue branches- Terpene synthase family-f genes. Purple branches- Terpene synthases that are generally expanded in *C. c. variegata*. Yellow branches- Terpene synthases that expanded after *C. c. variegata* diverged from *E. grandis*.

To estimate the relative ages of these gene family expansions, comparisons within eucalypt-specific, CCV-specific and *E.-grandis* specific expansions were investigated. First, CCV and *E. grandis* genes derived from 1:1:1 orthogroups among *Corymbia*:*Eucalyptus*:*Vitis* (outgroup) were used to visualize the Ks peak when *Corymbia*-*Eucalyptus* diverged. Then, eucalypt-specific (both CCV and *E. grandis*; *V. vitis* outgroup) expansions, CCV-specific expansions, and *E. grandis*-specific expansions were compared to find whether those expansions were relatively younger or older than the *Corymbia*-*Eucalyptus* split. The divergence peak among *Corymbia* and *Eucalyptus* genes occurs at Ks ~0.15 (Fig. 5a; Supplementary Data File 8). The eucalypt gene family expansions pre-date this divergence, with its main peak spread between Ks ~0.2–0.4 (total = 550; CCV = 265; *E. grandis* = 285). CCV-specific expansions displayed a bimodal peak, with some expansions occurring prior to divergence (Ks ~0.21; *n* = 101) and others undergoing a relatively recent expansion (Ks ~0.08; *n* = 132). These recent expansions suggest a dynamic mechanism for increasing gene numbers where function is enriched for sesquiterpenoid and triterpenoid biosynthesis (*P* = 0.02) as well as cutin, suberine, and wax biosynthesis (*P* = 0.03) (Supplementary Figs. 10–11; Supplementary Table 7). Plant cuticular wax compounds perform functions essential for the survival of terrestrial plants, including limiting non-stomatal water loss and gas exchange, protecting from ultraviolet radiation^77^, and forming physical barriers to herbivores and pathogens^78^. Eucalypts in particular require strong defense mechanisms to protect leaves during development, as young trees present large amounts of juvenile foliage making them a target for insect and fungal pests^79^. *Eucalyptus grandis* and CCV differ somewhat in wax morphology, with *E. grandis* exhibiting sparser wax coverage and irregular structure compared to *Corymbia*^80^. Concentrations of cuticular wax compounds in eucalypts have been linked to water loss response, for instance, a 10-fold increase in concentrations of n-alkane (wax compounds) was observed along an aridity gradient in both *Eucalyptus* and *Corymbia*^81^. Leaf wax and water repellency have also been linked to frost tolerance along an altitudinal cline in *Eucalyptus*^82^. The expansion in CCV genes linked to wax biosynthesis and the subsequent increase in concentration is likely selected for by the highly seasonal and variable rainfall environments occupied by CCV. However, the expansion of this gene family may be species- rather than genus-specific given the variation wax concentrations exhibited across species of *Eucalyptus* and *Corymbia*^81^.

Terpenes, in addition to regulating growth and developmental processes^83^, contribute chemical barriers to herbivory^84,85^, pollinator attraction,^86^, and thermotolerance^87^. The largest terpene synthase subfamily in CCV is TPS-a (responsible for sesquiterpene biosynthesis), which has undergone a recent expansion since diverging from *Eucalyptus* (Fig. 5b; Supplementary Data File 8). The TPS-b1 subfamily (responsible for monoterpene synthesis), was also generally expanded in *Corymbia*, based on the CCV-expanded orthogroups. The majority of terpenes synthesized by the two subfamilies are volatile and responsible for attracting pollinators and for plant defense via tritrophic interactions^88^. However, some sesquiterpenes are non-volatile phytoalexins that directly protect against fungal and bacterial pathogens^89^. Similarly, *E. grandis* specific expansions occurred post-divergence (Ks ~0.06; *n* = 296), with gene function enrichments related to sesquiterpenoid/triterpenoid biosynthesis (TPS-a) (*P* = 0.02) and glycan degradation (*P* = 0.03) (Supplementary Table 8). These CCV and *E. grandis* lineage-specific gene family expansions are likely due to selective pressures in environmental niches occupied by these two genera and appear to provide evidence of concerted evolution in eucalypts, but requires investigation of more species to lend support. In particular, genes involved in terpene biosynthesis have undergone separate but parallel expansions via tandem gene duplication^32^.

## Discussion

The generation of a high-quality *de novo* sequenced genome for *Corymbia citriodora* subsp. *variegata* has provided the opportunity to understand how evolutionary history has contributed to genome evolution within the Myrtaceae, an important and diverse group of angiosperms that have radiated across the Southern Hemisphere. After the gamma paleohexaploidy WGD (140 MYA) and divergence from other Rosids, Myrtales (the taxonomic order to which Myrtaceae belongs) underwent another lineage-specific WGD tetraploidy event (109 MYA). Paralogous sequence retention in the CCV genome underpins the importance of this event, finding functional enrichments for genes involved in pathogen inhibition, heat tolerance, and desiccation resistance, as well as pollinator attraction via unsaturated fatty acid metabolism and wax and terpene biosynthesis. This ancestral state of the eucalypt progenitor has been maintained even after *Corymbia* and *Eucalyptus* diverged, where large gene families responsible for mono- and sesqui-terpenes synthesis, leaf cuticle wax synthesis, and stress response pathways have expanded further. After the ancient tetraploidy event, most Myrtaceae underwent diploidization, and with few exceptions maintained a haploid chromosome number of eleven whilst exhibiting large differences in genome size (234–1110 Mb)^15^.

Within the eucalypts, *Corymbia* and *Eucalyptus* diverged ~60 MYA but nonetheless maintained synteny among their chromosomes despite also undergoing large chromosomal inversions (chromosomes 2,4,6,8,9,10 and 11) and translocations (chromosomes 2 and 6). Seventy-one percent of genes were captured in large syntenic blocks between the two genomes, with chromosome 3 being the most conserved. This conservation of chromosome 3 also extends to Northern Hemisphere *P. trichocarpa* (chromosomes XVIII and VI) and may be a result of the required synteny of conserved genes with related critical functions, which warrants further investigation. Throughout their evolutionary histories, both CCV and *Eucalyptus* have undergone gene family expansions whose function mainly relates to biotic and abiotic stress response. These gene family expansions have occurred in both separate (phenylpropanoid biosynthesis) and shared enzymatic pathways (cutin and terpenoid biosynthesis), while CCV also shows unique signatures of expanded key signaling components within the PTI pathway. While a number of these gene family expansions are shared, there is evidence of concerted and parallel evolution within CCV and *E. grandis* where gene families related to terpene biosynthesis (TPS-a) have expanded via tandem duplication in both species since they diverged.

The sequencing and description of the CCV genome will help inform future conservation efforts, molecular breeding, and global deployment of this taxa. In addition to Australia, *Corymbia* has been deployed to plantations in China, India, Sri Lanka, South Africa, Congo, and Kenya^90^. More recently, it has been successfully established in Brazilian plantations for hardwood, charcoal, and essential oil production and can outperform *Eucalyptus* in areas negatively impacted by climate-driven abiotic/biotic stresses^91^. *Eucalyptus* breeding programs and resources are well established, and knowledge regarding the stability of the *Corymbia* genome in terms of synteny, recombination, and tandem duplication will accelerate molecular breeding for both taxa and allow genomic resources already established for *Eucalyptus* (e.g. 60 K SNP chip, SSR markers)^92^ to be more easily transferable to other eucalypts. The release of additional Myrtaceae reference genomes will hopefully enable more extensive insights into evolutionary history based on comparative genomics across this important and diverse lineage of plants.

## Methods

### Illumina DNA library construction and sequencing

Genomic DNA was extracted^93^ from leaf tissue of *Corymbia citriodora* subsp. *variegata* genotype CCV2-018. Approximately 100 nanograms of DNA was sheared to 500 and 800 bp using the Covaris LE220 (Covaris), then size selected with SPRI beads (Beckman Coulter). DNA fragments were treated with end-repair, A-tailing, and ligation of Illumina compatible adapters (IDT, Inc) using the KAPA-Illumina library creation kit (KAPA biosystems). The prepared libraries (insert sizes 400 and 800 bp) were quantified using the next-generation sequencing KAPA Biosystem library qPCR kit, run on a Roche LightCycler 480 qPCR instrument. The two PCR-free Illumina libraries were multiplexed into pools then prepared for sequencing with a TruSeq paired-end cluster kit (v3) and Illumina cBot instrument to generate a clustered flowcell for sequencing. Sequencing of the flowcell was performed on the Illumina HiSeq2500 platform using a TruSeq SBS sequencing kit (v3) following a 2×250 indexed run recipe.

### PacBio library preparation and sequencing

Genomic DNA was sheared using the Covaris g-tube 20 Kb centrifugation protocol and purified using a 0.45X Ampure PB purification step. Single-stranded DNA fragments were removed using an exonuclease treatment, followed by DNA damage repair, end repair, and SMRTbell adapter ligation. In addition, a second exonuclease step removed failed ligation products (SMRTbell template prep kit 1.0). Ligated fragments were then the size selected for those >7 Kb in length (as short fragments are preferentially sequenced) then sequenced on the RSII instrument using P6-C4 chemistry and 4-h movie lengths. Reads were then processed using SMRTPortal (version 2.3.0) RS subreads protocol with default filtering settings (min subread length: 50; min polymerase read quality: 75; min polymerase read length: 50).

### RNA collection and sequencing

RNA was isolated from five tissue types: expanding and fully expanded leaves, flower buds and initials, and the outer chlorophyllous layer of bark cortex (Supplementary Fig. 3)^94^. Tissues were obtained from the CCV2-054 genotype (genetic map parent)^37^ and immediately preserved in a cryogenic shipping unit in the field for transport and storage prior to extraction. Total RNA was prepared using Ambion RNAquenous kit with Ambion RNA Isolation aid and the standard protocol (Life Technologies Australia Mulgrave Vic). Total RNA was shipped to AGRF (Melbourne, Australia) for library preparation (TruSeq® Stranded mRNA Sample, Illumina) and sequencing. A total of 75 Gb of RNA-seq was generated across all five libraries, 25 Gb of 100 bp single-end reads, and 50 Gb of 100 bp of paired end reads.

### Genome assembly

Contig assembly and initial scaffolding were conducted using Illumina paired-end reads. A total of 462,039,870 reads (representing ~163× genome coverage) were assembled Arachne (v.20071016)^35^, modified to handle larger datasets (data IO/sort functions/increased number of reads/alignments in memory). Arachne assembly parameters: macliq=800, remove_duplicate_reads=True, correct1_passes=1, BINGE_AND_PURGE_2HAP=True, max_bad_look=1000. Contig assembly and initial scaffolding steps produced 37,263 contigs into 32,740 scaffolds, totaling 563.0 Mb. Scaffold N50 length of the assembly was 132.6  Kb, with 1,430 scaffolds larger than 100 Kb. The resulting scaffolds were screened against bacterial proteins, organelle sequences, GenBank nr and removed if found to be a contaminant. In addition, scaffolds were removed if they were (a) repetitive, defined as scaffolds less than 50 Kb consisting of >95% 24 mers that occur four or more times in scaffolds >50 Kb, (b) contained only unanchored RNA, (c) <1 Kb in length, or (d) alternative haplotypes, defined as scaffolds <10 kb that align to scaffolds >10 Kb scaffolds with at least 95% identity and 95% coverage.

### PacBio patching

Gaps in the assembly were patched using ~25× sequencing coverage of PACBIO filtered subreads. Gaps were patched by first breaking scaffolds into contigs. Contigs <1 Kb were excluded from the gap patching process. Subsequently, 1 Kb of the sequence was trimmed off the contig ends and the trimmed portion was broken into 100 mers. The 100 mers were aligned to the PACBIO reads using the short read aligner bwa^95^, and individual PACBIO reads were mapped to scaffolds indicated by the 100mer alignments. QUIVER was used to assemble gap crossing reads for gaps with more than 5 filtered subreads crossing them. The resultant assembled sequence was used to patch the gap. A total of 3,149 gaps were patched, with a total loss of 55,511 bases from the raw assembly due to the presence of negative gaps in the assembly. Mis-assemblies were assessed by identifying gaps where 5 or more PACBIO reads have >1 Kb regions of the read aligning to two different scaffolds. A total of 166 mis-joins were identified and the breaks made, with the associated join being made using the reads that indicated a break. A total of 485 additional joins were made using the PACBIO reads. Additional scaffolding of the genome was performed using SSPACE-Standard (Version 2.0) with Nextera long mate pair prepared libraries (Insert size 4 and 8 Kb). SSPACE scaffolding was performed using default parameters and no extension (*x* = 0).

### Anchoring scaffolds to linkage maps

The retained assembly from the gap patched assembly and SSPACE scaffolding were anchored into pseudomolecules using the ALLMAPs pipeline^39^. Three individual genetic maps were generated representing each parent of the two pedigrees (male map- CCV2-054, female maps- CT2-050 and CT2-018). The use of a ‘marker binning’ procedure, and stringent criteria for the inclusion of markers, resulted in robust marker orders in the linkage maps evidenced by high rank-order correlations among shared markers^37^. The sequence of each marker was used to anchor (and orient, if a second marker was available) contigs with matching sequence onto specific linkage groups, with a greater weighting given to the order of markers from CCV.

ALLMAPs incorporates a methodology for computing a scaffold order that maximizes collinearity across a collection of maps and generates outputs of ordered and orientated scaffolds. The pipeline was run using the default settings, except that filtering was applied so that linkage groups with <20 markers were removed from the analysis, joins between scaffolds were padded with 100 N, and a weighting of 1 (i.e., highest confidence of marker order) was applied to the CCV2-054 map, and a weighting of 2 was applied to both of the *C. torelliana* maps. A lower weighting applied to the *C. torelliana* was used to allow for a higher likelihood of the possibility of marker reordering in the nonfocal species. Similarity searches aimed at matching DArT-seq markers sequence tags from the genetic maps with scaffolds were determined by using the blastn program from BLAST^96^ where a threshold *e*-value of 1 × 10^−10^ was used as a cutoff and only the best match was taken. Chromosomes and subsequent tracks in Fig. 1 were created using Circa (http://omgenomics.com/circa).

### Single-copy gene analysis

*Eucalyptus grandis* protein sequences were re-aligned to the *E. grandis* genome sequence using BLAT^97^ (-noHead -extendThroughN t=dnax q=prot) to find single-copy genes sequences. 20,256 single-copy genes were identified within *E*. *grandis* (no tandem duplications, no gene splice variants, 90% gene coverage, 85% gene identity, >300 bp). Aligned to CCV (using the same BLAT parameters), 16,245 proteins were found similar (>75% ID; 90% coverage), of which 14,911 were also present in single copy (92%) in the CCV assembly and of those 90% were present on pseudomolecules.

### Protein-coding gene classification and annotation

Adequate RNA mapping for transcript assembly and protein prediction was verified (88% average mapping across tissues), as well as global sequence identity between genotypes (97% global; 98% CDS). Transcript assemblies were made from ~260 M pairs of 2 × 100 stranded paired-end Illumina mRNA-seq reads using PERTRAN as used in other plant genome annotations^98^. 99,336 transcript assemblies were constructed using PASA^99^ from mRNA-seq transcript assemblies above. Loci were determined by transcript assembly alignments and/or EXONERATE alignments of proteins from arabi (*Arabidopsis thaliana*), soybean, poplar, tomato, Kitaake rice, brachy, aquilegia, eucalyptus, grape, and Swiss-Prot proteomes to the repeat-soft-masked genome assembly using RepeatMasker^100^ with up to 2 Kb extension on both ends unless extending into another locus on the same strand. Repeat library consists of *de novo* repeats by RepeatModeler^101^ on the CCV genome. Gene models were predicted by homology-based predictors, FGENESH+^102^, FGENESH_EST (similar to FGENESH+, EST as splice site and intron input instead of protein/translated ORF), and GenomeScan^103^, PASA assembly ORFs (in-house homology constrained ORF finder) and from AUGUSTUS via BRAKER1^104^. The best-scored predictions for each locus were selected using multiple positive factors including EST and protein support, and one negative factor: overlap with repeats. The selected gene predictions were improved by PASA. The improvement includes adding UTRs, splicing correction, and adding alternative transcripts. PASA-improved gene model proteins were subject to protein homology analysis to above-mentioned proteomes to obtain Cscore and protein coverage. Cscore is a protein BLASTP score ratio to MBH (mutual best hit) BLASTP score and protein coverage is the highest percentage of protein aligned to the best of homologs. PASA-improved transcripts were selected based on Cscore, protein coverage, EST coverage, and its CDS overlapping with repeats. The transcripts were selected if its Cscore was larger than or equal to 0.5 and protein coverage larger than or equal to 0.5, or it had EST coverage, but its CDS overlapped with repeats less than 20%. For gene models whose CDS overlaps with repeats for more than 20%, its Cscore was at least 0.9 and homology coverage was at least 70% to be selected. The selected gene models were subject to Pfam analysis and gene models whose proteins were more than 30% in Pfam. TE domains were removed along with weak gene models. Incomplete gene models, models where there was low homology support without fully transcriptome support, short single exons (<300 BP CDS) without a protein domain, or a lack of good expression gene model, were manually filtered out.

### Syntenic blocks

All pairwise BLAST hits were calculated with Diamond^105^ either separately, or within the Orthofinder^106^ program. Hits were then culled to the top two hits within each haplotype (so, if a diploid is mapped to a diploid, four hits would be retained for each gene; if it were mapped to a tetraploid, eight hits would be retained). All hits with a bit score <50 were dropped.

Initial orthogroup inference: A separate run of Orthofinder was made for each pair of genomes using the culled BLAST results. Only those hits within ‘orthogroups’ were retained.

Initial block construction: Blocks were formed using MCScanX from the culled and orthogroup-constrained BLAST results, allowing 5× as many gaps in the alignment as 50% of the minimum block size (MBS, default = 10). Block were then pruned with DBSCAN to blocks with MBS hits within a fixed gene-rank radius 5× MBS. All orthogroups are then ‘completed’, where igraph expanded the orthogroups to include all possible combinations among genomes. These blast hits were then pruned with dbscan with identical parameters as above. Block cleaning and extension: To fill potential gaps in blocks left by the stochastic nature of varying orthogroup connectivity, we pull all blast results that passed the score threshold (agnostic to orthogroup identity) that were within a 100-gene radius of any syntenic block and re-form blocks with MCScanX with the same parameters as above.

Syntenic orthology inference: All BLAST results were culled to those within a 50-gene rank radius of any syntenic block for all genomes. Orthofinder was run on this entire set, and  BLAST hits were parsed into orthologs, paralogs, or un-clustered homologs. By default, hits that were not in an orthogroup (neither orthologs or paralogs) with a score <50 or <50% of the best bit score for that gene -by- unique genome combination were dropped from this dataset.

### Gene family analysis and mutation rate

Single-copy gene orthologs and gene family expansions were characterized using Orthofinder (v2.2.7)^106^. All on all protein sequences from *C. citriodora*, *E. grandis*, *S. purpurea*, *P. trichocarpa,* and *V. vinifera* were performed using Diamond^105^, then clustered into orthogroups using Orthofinder. Single-copy orthologs were determined as clusters containing one protein sequence from each of the five species. Protein alignments among species were performed using MAFFT (v7.464)^107^ and the coding sequence was extracted using a pal2nal^108^ perl script. Pairwise synonymous mutation rates were calculated from coding sequences using PAML^109^ codeml. Ks mutation rate/site/year (R) was calculated as: R = Ks/(2 * divergence age). Estimates of population mutation rate (4N_e_µ) was obtained from the CCV parental library and four unrelated genotypes (CCV2-019;−025;−045;−046) following maximum likelihood estimators based on the alignment of shotgun sequence data^110^ to the CCV genome sequence, using bwa mem (version 0.7.17-r1188)^95^. Upper and lower confidence bounds of estimates per chromosome were extracted per diploid genotype. At each position in the chromosome we count the four different nucleotides, n: = (nA,nC,nG,nT), and such a quartet of counts was called a profile, while the sum of counts, n = nA + nC + nG + nT, was the coverage of the profile’s position. Heterozygous profile position were taken as those passing SNP calling using the GATK (version 3.8) program^111^ following the best practices pipeline^112^, while homozygous profile positions were taken using the minimum depth of 4. The neutral mutation rate (µ) per base pair per generation (year) was re-estimated based on these estimates of population mutation rate from shotgun data, assuming a generation time of 15 years in CCV and an ancestral population size equals to 112,421, which was suggested previously to be consistent with the demographic past of the related species of *E. grandis*^46^.

### Paralogs and whole-genome duplication

Paralogs among *C. citriodora*, *E. grandis*, and *V. vinifera* (outgroup) were extracted from orthogroup and ortholog information generated from Orthofinder. Pairwise Ks substitution rates among paralogs for each species were calculated using codeml. *Corymbia* paralog gene pairs underlying the eucalypt specific peak (0.33–0.45) were extracted and investigated for GO and KEGG pathway enrichment.

### Differential tissue expression

Tissue-specific RNA libraries were aligned to the indexed *Corymbia* genome using STAR^113^ (v2.5.3a; parameters: -outFilterMultimapNmax 7 -outFilterMismatchNmax 4). Gene count tables were exported and analyzed using the edgeR package^114^ to obtain reads per kilobase million (rpkm) expression values for each tissue. Normalized gene counts were verified using a heatmap to ensure expression among related tissues was consistent.

### Gene family expansions

Eucalypt, *Eucalyptus,* and *Corymbia* specific gene family expansions (as defined in “Results”) were isolated from Orthofinder orthogroups. Pairwise Ks values among sequences were calculated using codeml within each of the three classes of gene family expansion to investigate which families had accumulated relatively more or less synonymous mutations than *Corymbia*-*Eucalytpus* single-copy orthologs. Terpene genes were defined using best BLAT^97^ hits among *Corymbia* proteins to sequences from Kulheim et al.^33^. The unrooted terpene gene family tree was generated using FastTree^115^ (v2.1.10) and visualized using iTOL^116^, from MAFFT^107^ aligned *Corymbia* terpene transcript sequences.

### GO and KEGG pathway enrichment analysis

Gene ontology (GO) enrichment analysis was carried out using topGO, an R Bioconductor package^117^ with Fisher’s exact test; only GO terms with a *P* < 0.05 were considered significant. To identify redundant GO terms, semantic similarity among GO terms were measured using Wang’s method implemented in the GOSemSim, an R package^118^. KEGG^119^ pathway enrichment analysis was performed based on hypergeometric distribution test and pathways with *P* < 0.05 were considered enriched.

### Reporting summary

Further information on research design is available in the Nature Research Reporting Summary linked to this article.

## Supplementary information

Supplementary Information

Description of Additional Supplementary Files

Supplementary Data File 1

Supplementary Data File 2

Supplementary Data File 3

Supplementary Data File 4

Supplementary Data File 5

Supplementary Data File 6

Supplementary Data File 7

Supplementary Data File 8

Reporting Summary

## Data Availability

Additional work to support the findings of this manuscript can be found in the supplementary data section. Raw Illumina (paired end and mate pair) and PacBio reads are available from the National Center for Biotechnology Information Short Read Archive (SRA) under accession: PRJNA234431. Additional CCV resequencing genotypes are available from SRA under accessions: PRJNA333377, PRJNA333376, PRJNA333375, PRJNA333374. Illumina RNASeq data are available at NCBI under BioProject: PRJNA629009. The genome assembly and annotation are freely available at Phytozome (https://phytozome-next.jgi.doe.gov/info/Ccitriodora_v2_1). This Whole Genome Shotgun project has been deposited at DDBJ/ENA/GenBank under the accession JABURB000000000. The version described in this paper is version JABURB0100000000. All relevant data are available upon request from the corresponding author (Adam Healey).
