# Supplementary material for: Pests, diseases, and aridity have shaped the genome of Corymbia citriodora
Source: Commun Biol. 2021 May 10;4:537. doi: 10.1038/s42003-021-02009-0 (PMC8110574; doi:10.1038/s42003-021-02009-0)
Supplement: Supplementary file 1 — Supplementary Information [file 42003_2021_2009_MOESM1_ESM.pdf]

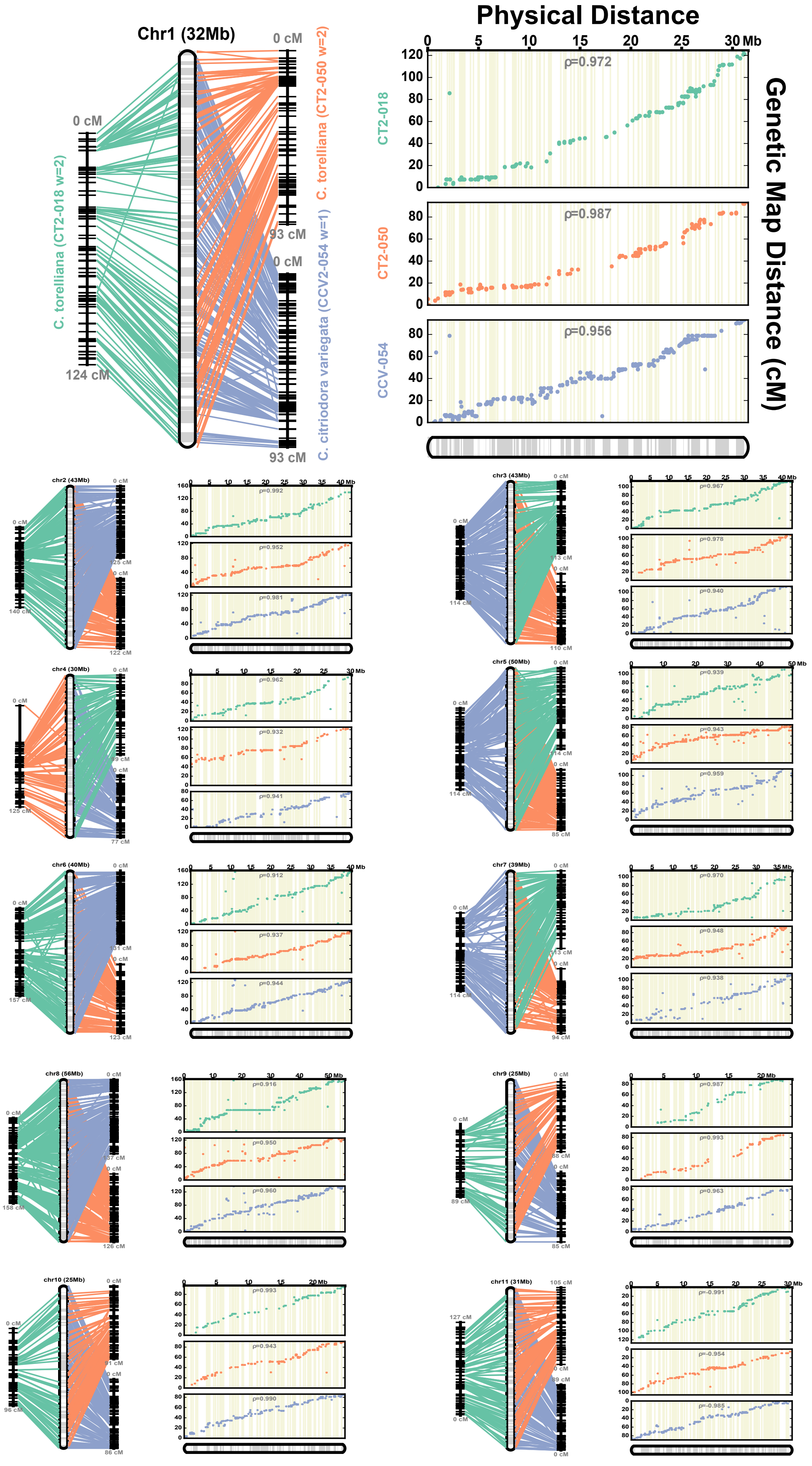

Supplemental Figure 1-ALLMAPs chromosome construction from *Corymbia* genetic map markers. Chromosome builds (1-11) were generated from the consensus of three *Corymbia* genetic maps derived from controlled crosses between *C. torelliana* (genotypes CT-018/CT2-050) and *C. citriodora variegata* (CCV2-054). The greatest weight for ordering scaffolds was given to the CCV genetic map where there were marker order conflicts. Average Spearman correlation coefficient for all three maps across 11 chromosomes was r = 0.96.

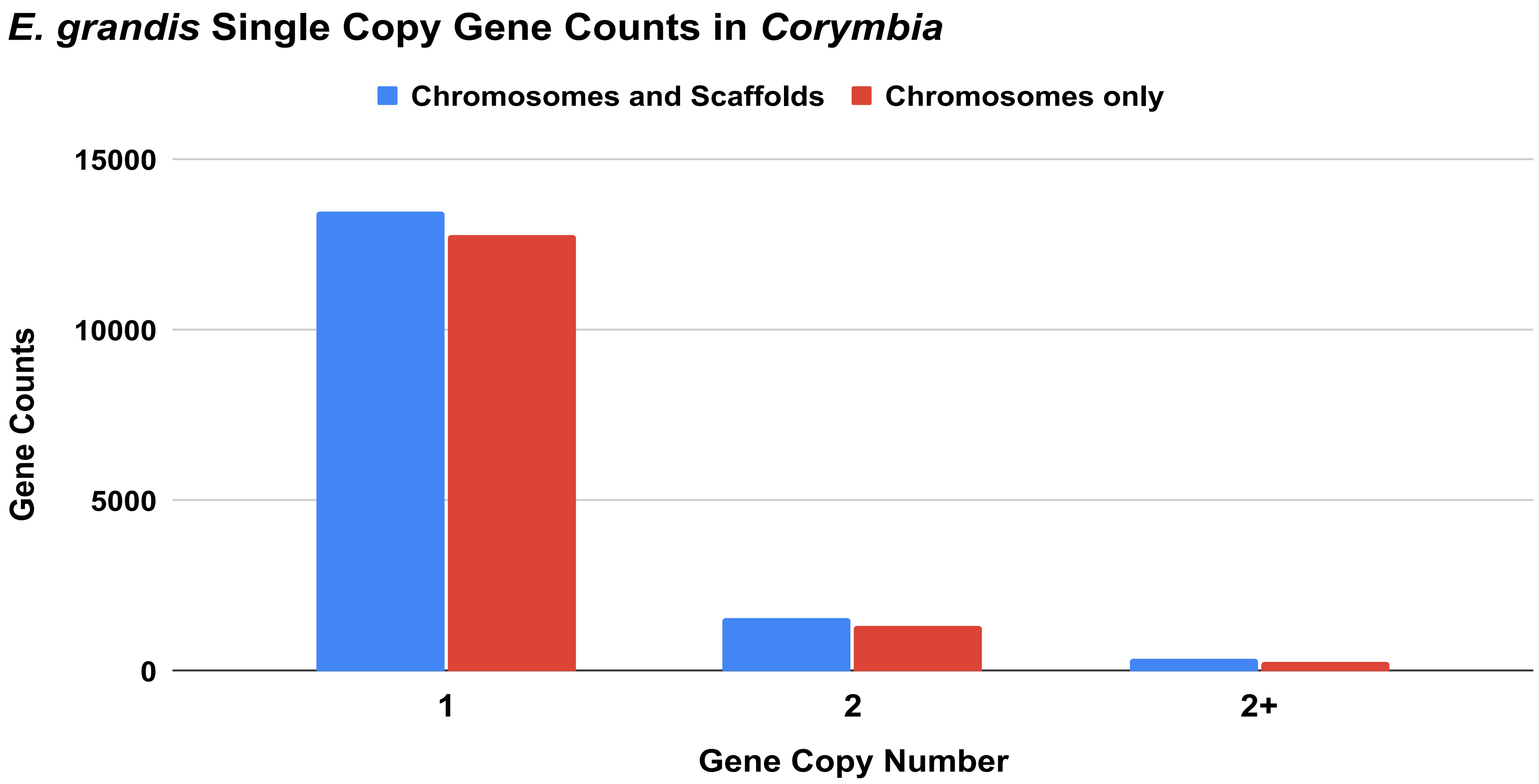

Supplemental Figure S2- *Eucalyptus grandis* single copy genes aligned to *Corymbia citriodora* subspecies *variegata*. Single copy proteins (n= 20,256) were extracted from *E.grandis* (primary protein sequence, 90% gene coverage, 85% gene identity, >100 amino acids, single alignment) and aligned to *C.c. variegata.* Alignments were considered if they passed filtering criteria (>75% identity; >=90% coverage). Of those genes that passed the filtering criteria (16,207), 14,911 were present in single copy (92%) and 90% were located on chromosomes.

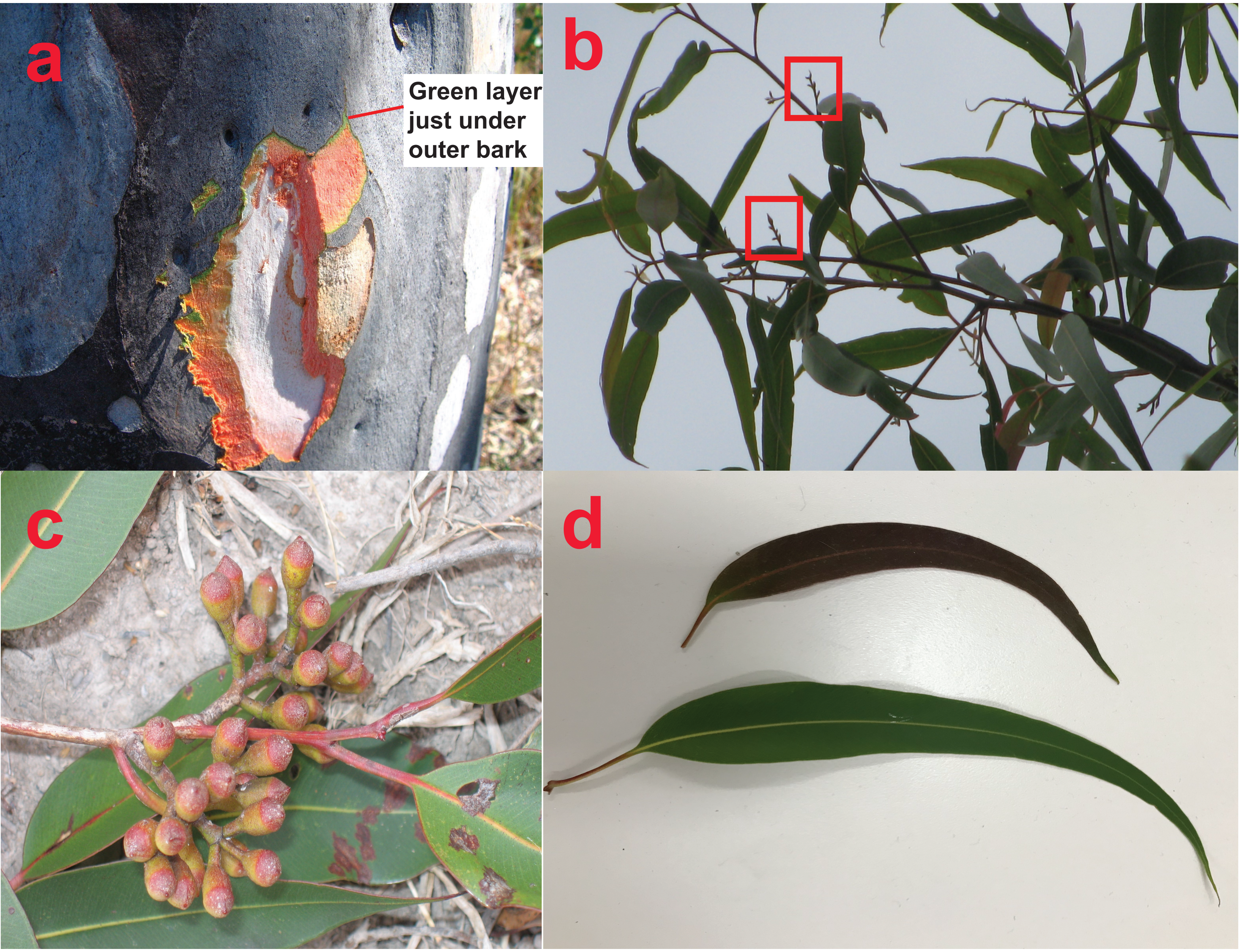

Supplemental Figure 3- *Corymbia citriodora* subspecies *variegata* tissues used for RNA extraction.
a) Photosynthetic bark cortex: outer bark is removed and chlorophyllous (green) layer is collected.
b) Flower initials (shown in rectangles) c) flower buds d) expanding (top) and fully expanded (bottom) leaves.

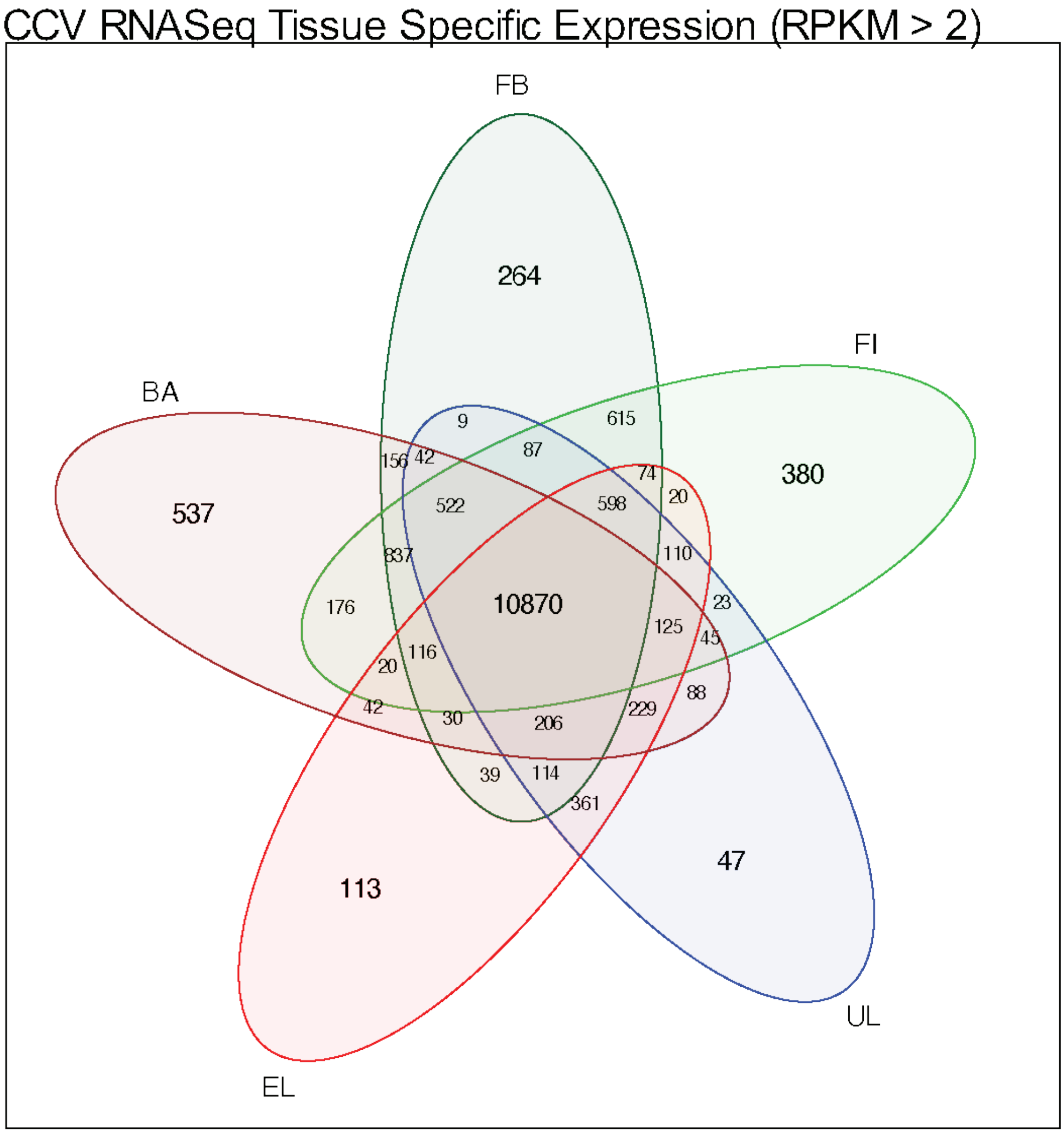

Supplemental Figure 4- Tissue specific expression of *Corymbia citriodora subsp. variegata* genes. RNA libraries collected from photosynthetic bark cortex (BA), unexpanded leaves (UL), expanded leaves (EL), flower buds (FB) and flower initials(FI) were mapped onto the *Corymbia* reference genome using STAR Aligner. Gene counts were calculated from each library and converted to reads per kilobase million (RPKM) using Bioconductor package edgeR.

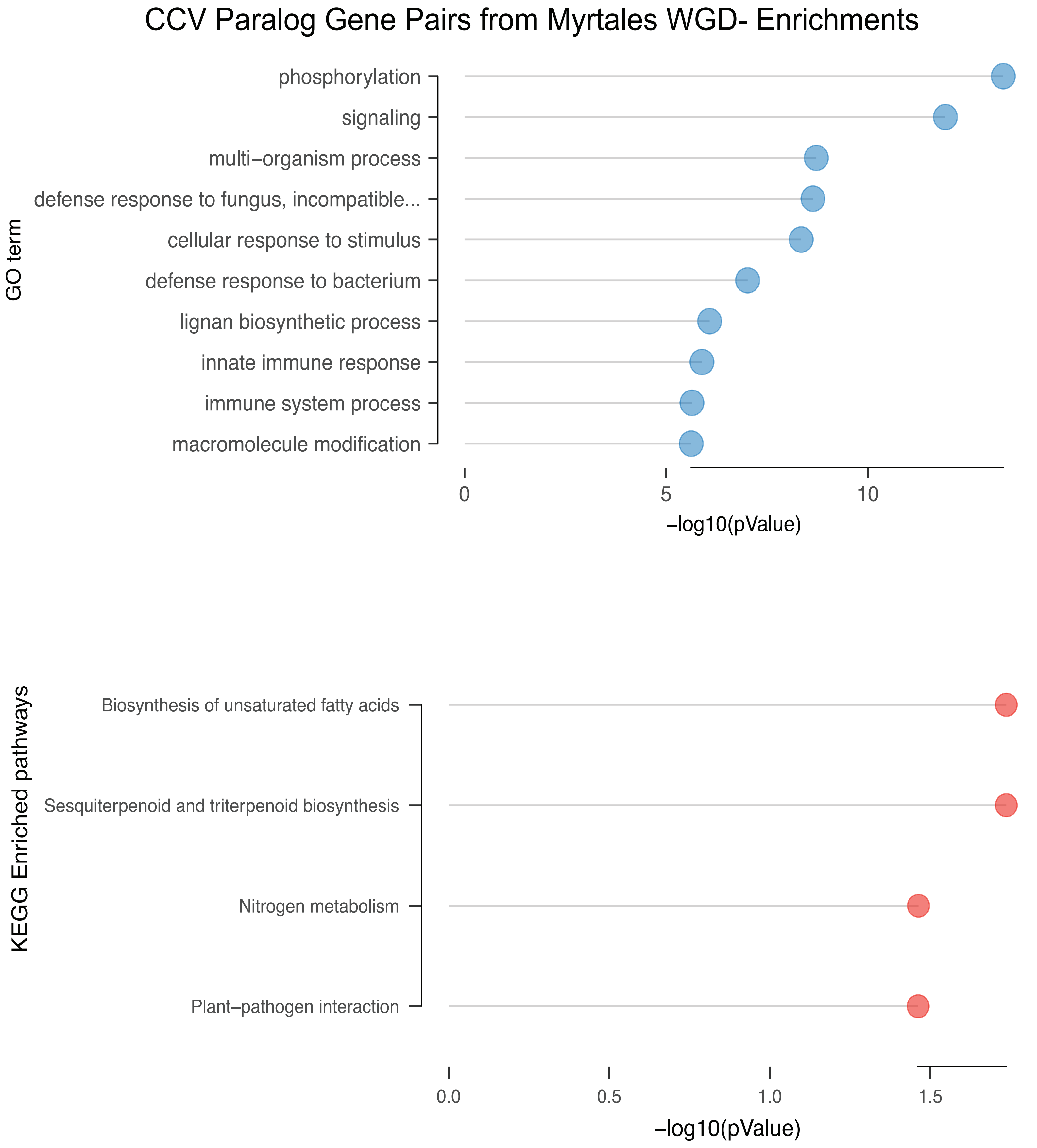

Supplemental Figure S5- GO Term and KEGG pathway enrichments for *Corymbia citriodora subspecies variegata* paralog gene pairs derivedfrom the Myrtales specific whole genome duplication (Ks peak: 0.27-0.40; total gene number= 528).

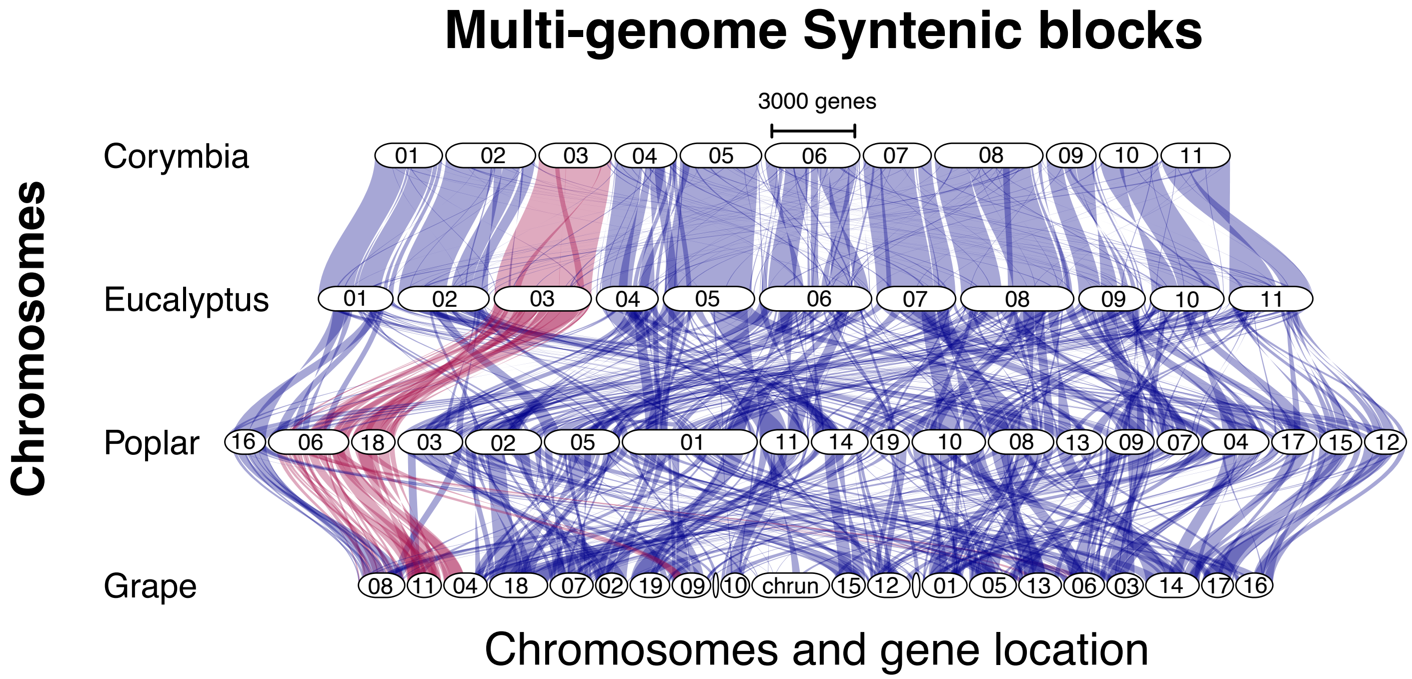

Supplemental Figure S6 - Multi-genome syntenic blocks among *Corymbia*, *Eucalyptus*, Poplar and Grape genomes. Gene blocks originating from *Corymbia* chromosome 3 are highlighted and tracked throughout each proceding genome.
Corymbia- *Corymbia citriodora* subspecies *variegata*; *Eucalyptus*- *Eucalyptus grandis*; Poplar- *Populus trichocarpa*; Grape- *Vitis vinifera*.

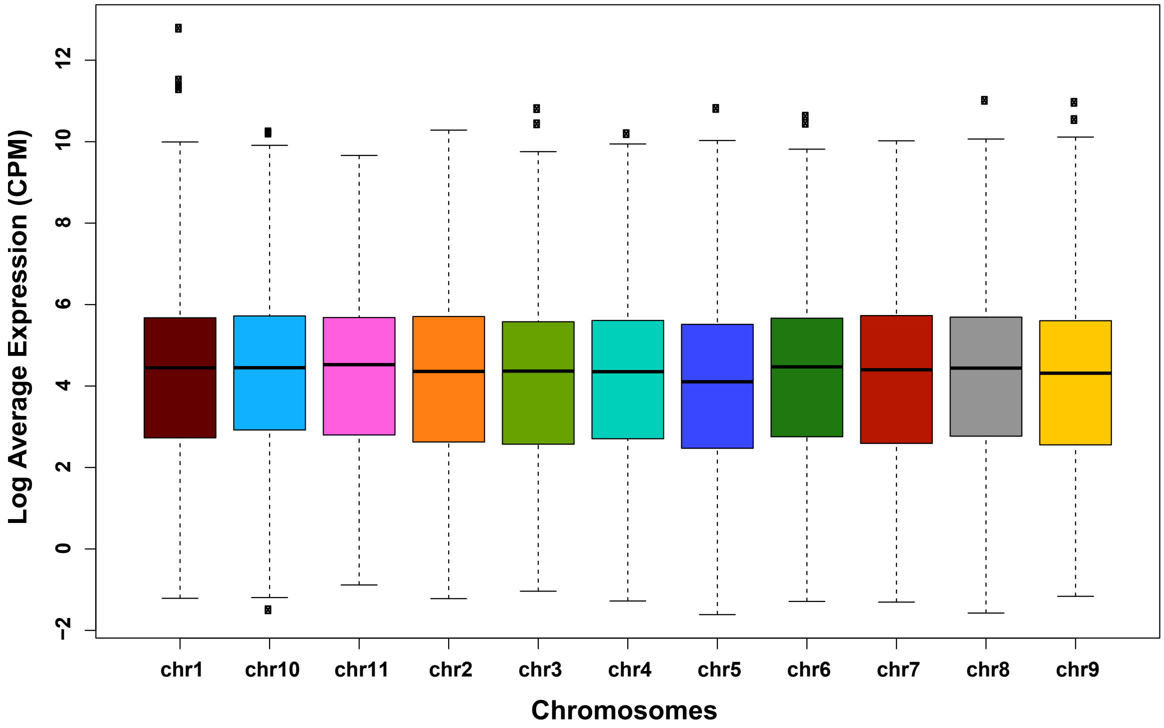

Supplemental Figure S7- Median expression among chromosomes from *Corymbia citriodora* subspecies *variegata* sample tissues. RNA libraries collected from photosynthetic bark cortex, unexpanded Leaves, expanded Leaves, flower buds and flower initials were mapped onto the CCV reference genome using STAR Aligner. Gene count averages were calculated from each library and converted to log average counts per milliontranscripts (cpm) using Bioconductor package edgeR. The black bar within each box represents the median gene expression value for all genes on that chromosome. Each box represents the interquartile range with the whiskers showing the highest and lowest values.

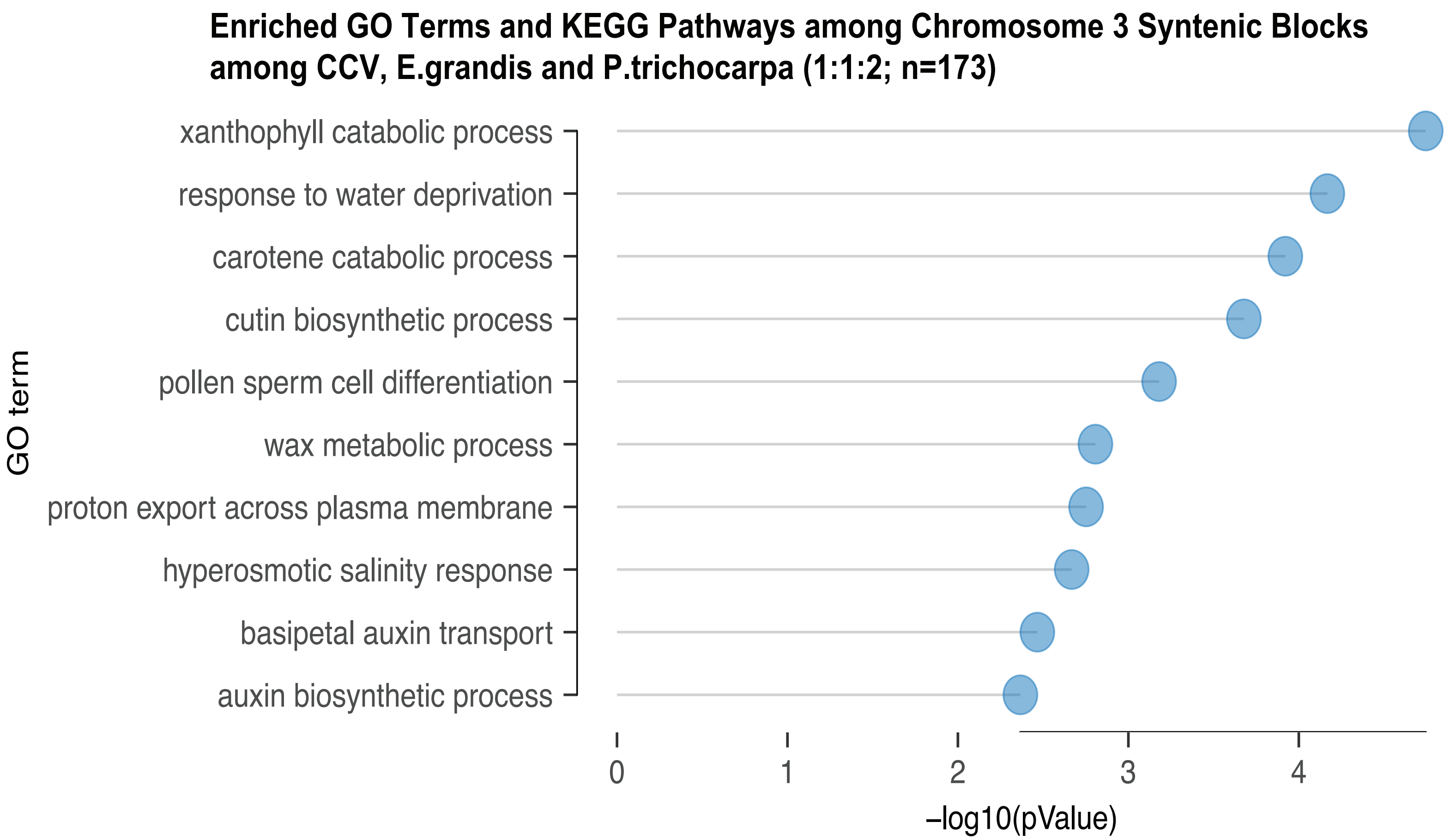

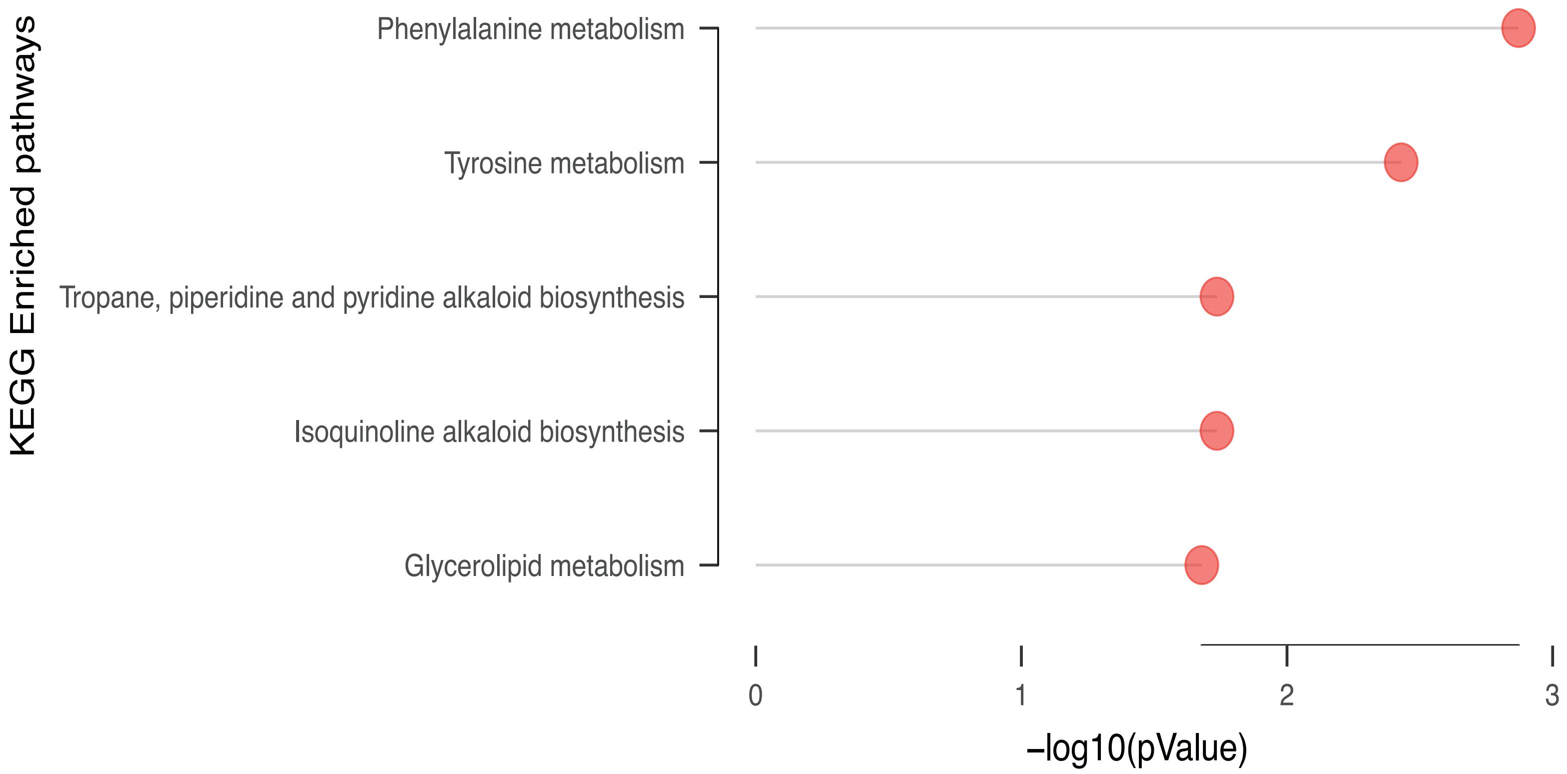

Supplemental Figure 8- KEGG pathway enrichments among chromosome 3 syntenic blocks between *Corymbia*, *Eucalyptus* and Poplar. Enrichment (p<0.05; unadjusted) was constrained to gene blocks with a 1:1:2 relationship among *Corymbia/Eucalytpus* chromosome 3 and chromosomes VI and XVIII in *P. trichocarpa*.

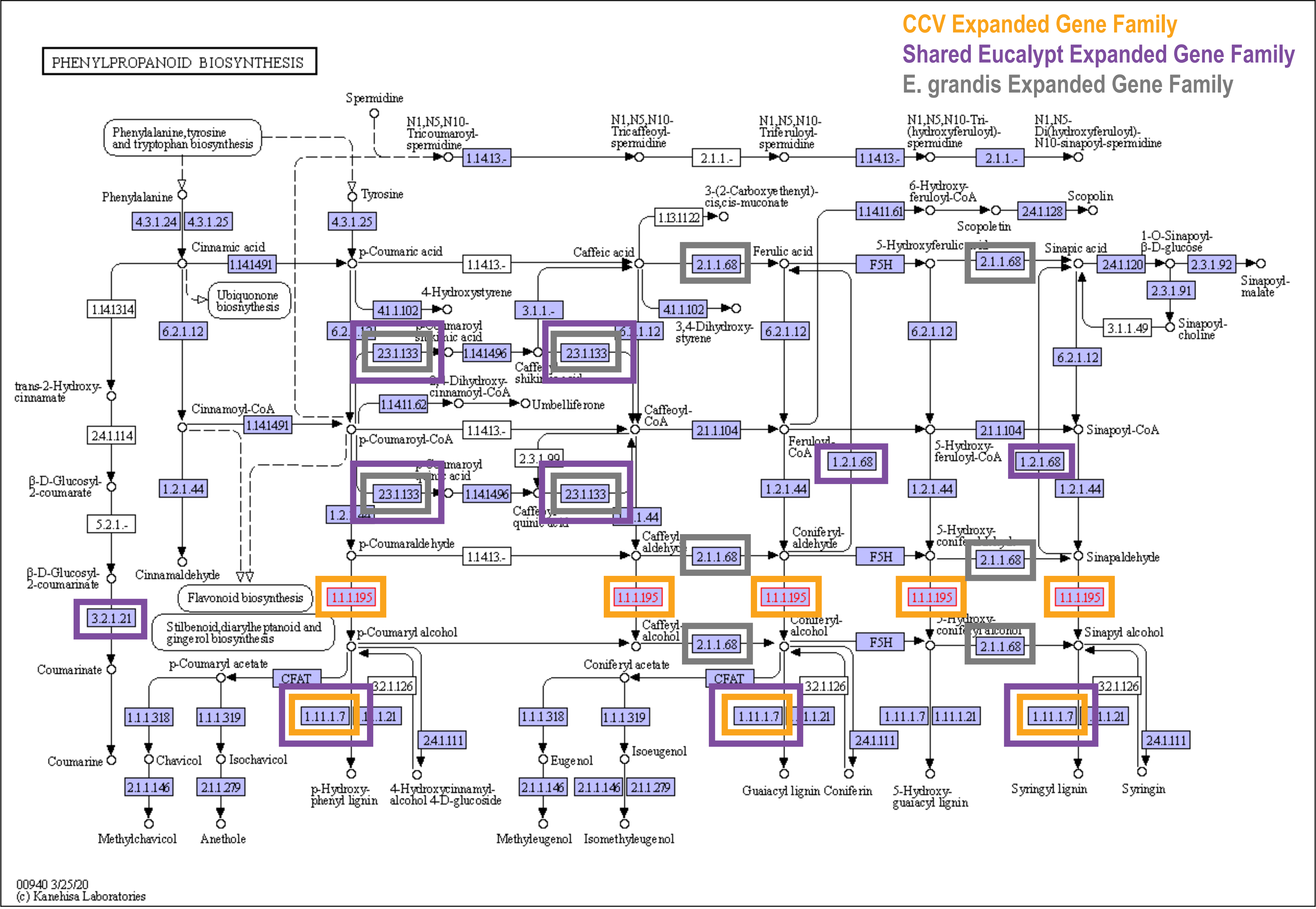

Supplemental Figure 9- Significant gene family expansions that occur in the phenylpropanoid pathway (map 00940). Genes belonging to the pathway that were significantly enriched among shared eucalypt, CCV-specific and *E. grandis*-specific expansions were mapped onto the pathway and highlighted. CCV- *Corymbia citriodora* subspecies *variegata*

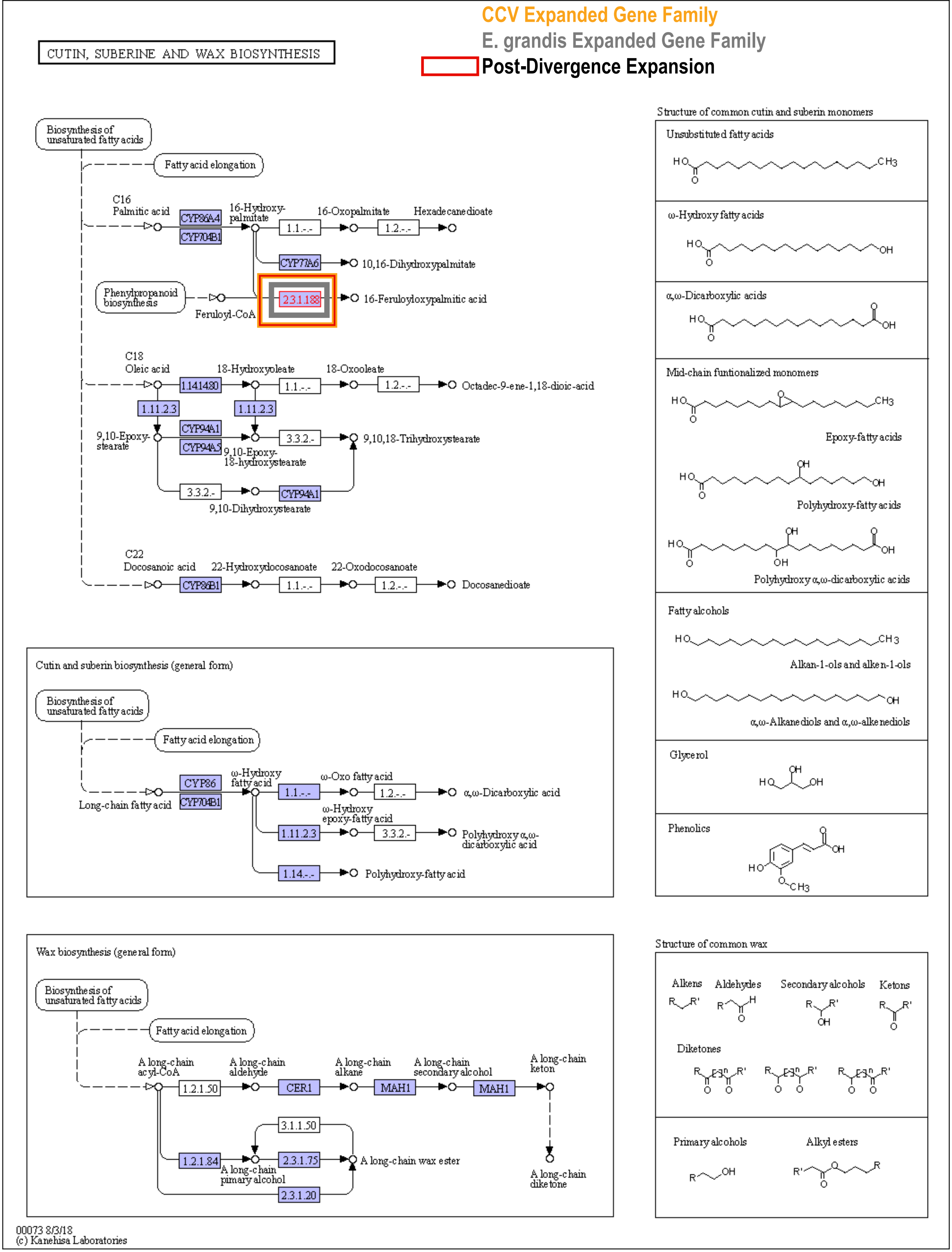

Supplemental Figure S10- Significant gene family expansions that occur in the cutin, submarine and wax biosynthesis pathway (map 00073). Genes that were significantly enriched among CCV-specific and *E. grandis*-specific expansions were mapped onto the pathway and highlighted.
Gene families which also appear to have expanded post-divergence between *Corymbia* and *Eucalyptus* are marked with a red rectangle. CCV- *Corymbia citriodora* subspecies *variegata*

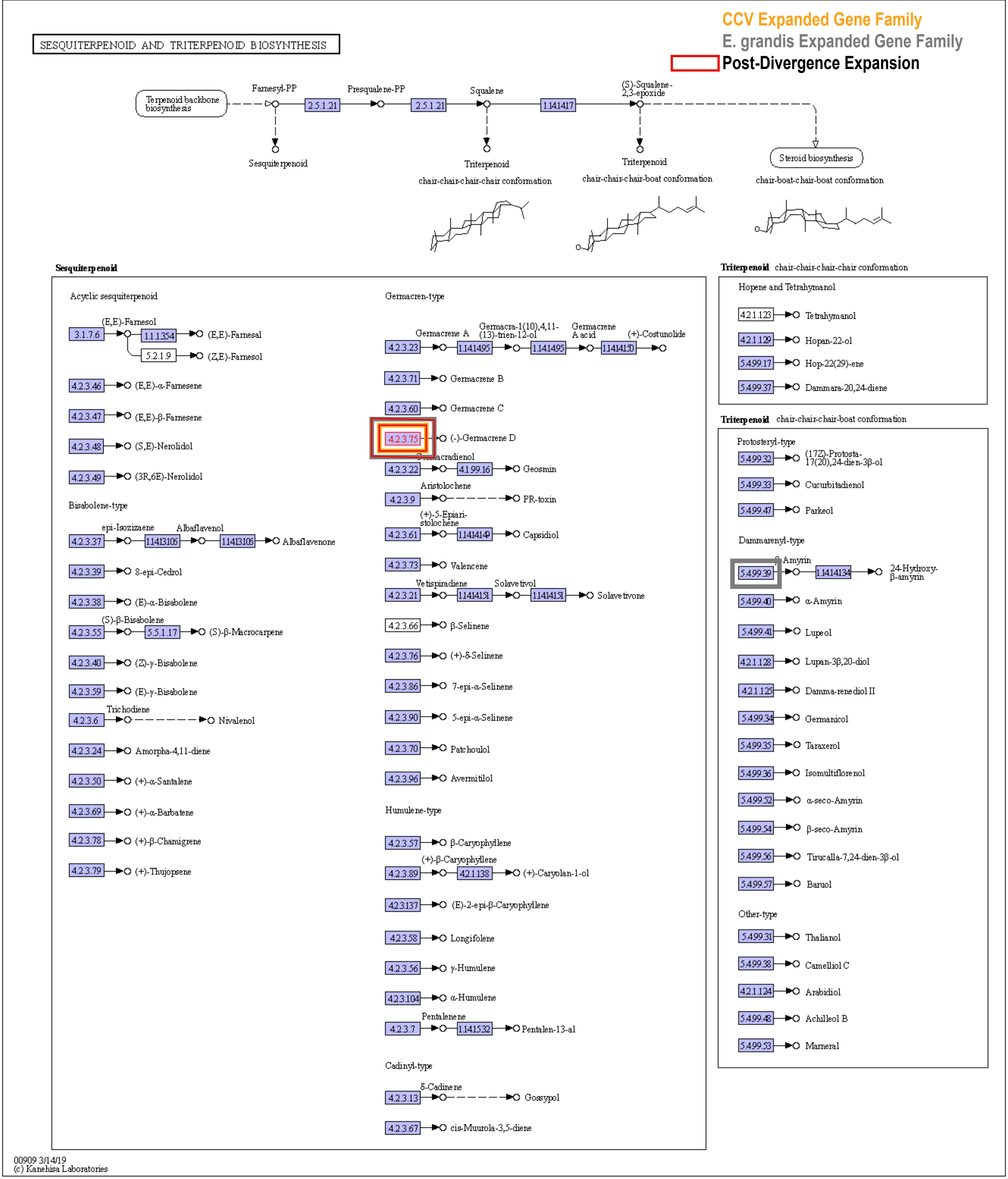

Supplemental Figure S11- Significant gene family expansions that occur in the sesquiterpenoid and triterpenoid biosynthesis pathway (map 00909). Genes that were significantly enriched among CCV-specific and *E. grandis*-specific expansions were mapped onto the pathway and highlighted.
Gene families which also appear to have expanded post-divergence between *Corymbia* and *Eucalyptus* are marked with a red rectangle. CCV- *Corymbia citriodora* subspecies *variegata*

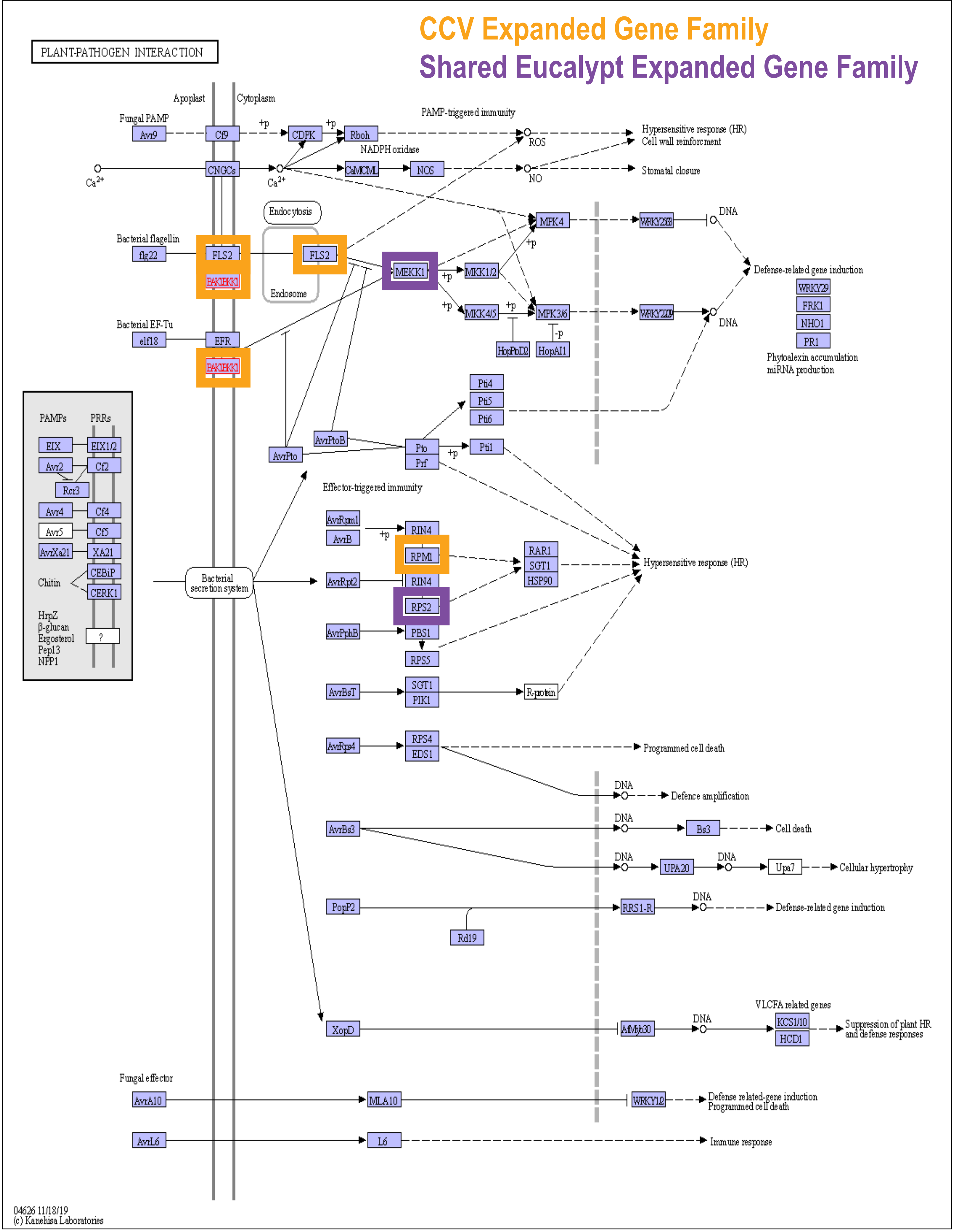

Supplemental Figure 12- Significant gene family expansions that occur in the plant-pathogen interaction pathway. Genes that were significantly enriched among shared eucalypt and CCV-specific expansions were mapped onto the pathway and highlighted. CCV- *Corymbia citriodora* subspecies *variegata*

Supplemental Table 1- Benchmarking Universal Single-Copy Orthologs (BUSCO v3.0.2; embryophyta_odb9 database) score for the Corymbia citriodora subspecies variegata genome annotation.

	Chromosomes and Scaffolds	Chromosomes Only
Percent complete BUSCOs	95.1	89.6
Percent complete single copy BUSCOs	89	85.6
Percent complete duplicated BUSCOs	6	4
Percent fragmented BUSCOs	2.8	2.9
Percent missing BUSCOs	2.1	7.5

Supplementary Table 2: Average Recombination Rates per Chromosome in *C.c. variegata* . Recombination rates were calculated using DArT-Seq markers from Butler et al. 2017

Chromosome	Recombination rate (cM/MB)
1	2.95
2	2.93
3	2.67
4	2.55
5	2.39
6	3.27
7	2.97
8	2.47
9	3.44
10	3.51
11	2.95
Genome -wide	2.85

Supplementary Table 3- Average Number of crossover (CO) events per *C.c. variegata* chromosome. Correlation between CO's and chromosome size is 0.92.

	Average CO	Chr length (MB)
Chr1	1.03	31.55
Chr2	1.41	42.9
Chr3	1.33	42.68
Chr4	0.95	30.21
Chr5	1.34	50.16
Chr6	1.48	40.21
Chr7	1.35	38.6
Chr8	1.71	55.75
Chr9	0.91	24.78
Chr10	0.99	24.99
Chr11	1.01	30.79

Supplementary Table 4-KEGG pathway enrichments for shared eucalypt (both C.c.variegata/E.grandis) gene family expansions. Only orthologs within C.c.variegata are provided.
Expansions are defined as orthogroups containing 5 or more genes and more than 70% of which are derived from both E.grandis and C.c.variegata.

Pathway	PathwayID	KO.ratio	Odds ratio	Expected	Pvalue	GeneID
Phenylpropanoid biosynthesis	egr00940	4/20	24.35	0.22517321	5.8435E-05	Cocit.G0313;Cocit.G0314;Cocit.C2226;Cocit.C2228;Cocit.C2230;Cocit.C2264;Cocit.B2086;Cocit.B2087;Cocit.H0612;Cocit.H0614;Cocit.J0426;Cocit.J0427
Cyanoamino acid metabolism	egr00460	2/11	20.5165165	0.12384527	0.00637248	Cocit.I0116;Cocit.I0117;Cocit.I0198;Cocit.I0200;Cocit.J0426;Cocit.J0427
Pentose and glucuronate interconversions	egr00040	2/15	14.1871102	0.16887991	0.01182508	Cocit.B2295;Cocit.F0351;Cocit.F0353;Cocit.F0354
Isoflavonoid biosynthesis	egr00943	1/2	90.1052632	0.02251732	0.02239378	Cocit.H2513;Cocit.H2514
Monoterpenoid biosynthesis	egr00902	1/3	45.0394737	0.03377598	0.03340667	Cocit.F3169;Cocit.F3170;Cocit.F3175
Glucosinolate biosynthesis	egr00966	1/3	45.0394737	0.03377598	0.03340667	Cocit.I0116;Cocit.I0117;Cocit.I0198;Cocit.I0200
Plant-pathogen interaction	egr04626	2/30	6.55791506	0.33775982	0.04407368	Cocit.G0179;Cocit.G0181;Cocit.G0193;Cocit.G0196;Cocit.G0431;Cocit.G0433;Cocit.F1916;Cocit.F1919;Cocit.F1924;Cocit.D0101

Column	Description
KO.ratio	Number of KEGG Orthology terms in geneset/number of KEGG Orthology terms annotated in the pathway)
Odds.ratio	The strength of association between geneset and annotated pathway
Expected	Expected number of terms in the geneset
P-value	P-value, Hypergeometric test

Supplemental Table 5- KEGG pathway enrichments for E. grandis specific gene family expansions.
Expansions are defined as orthogroups containing 5 or more genes and more than half of which are derived from E.grandis.

Pathway	PathwayID	KO.ratio	Odds ratio	Expected	Pvalue	GeneID
Galactose metabolism	egr00052	3/17	49.1020408	0.08922507	7.8086E-05	Eucgr.L00251;Eucgr.L00240;Eucgr.L00249;Eucgr.L00234;Eucgr.L00243;Eucgr.B01793;Eucgr.L00235;
						Eucgr.L00250;Eucgr.L00245;Eucgr.B01791;Eucgr.L00248;Eucgr.L00241;Eucgr.H00549;Eucgr.K01950;Eucgr.K01957;Eucgr.K01949;Eucgr.K01960;Eucgr.K01962;Eucgr.K01956
Pentose and glucuronate interconversions	egr00040	2/15	38	0.06946588	0.00203046	Eucgr.H00549;Eucgr.H03295;Eucgr.H03296;Eucgr.L00257;Eucgr.H03297;Eucgr.B01686
Flavonoid biosynthesis	egr00941	2/16	32.744898	0.07903674	0.00263732	Eucgr.G01530;Eucgr.G01531;Eucgr.G01532;Eucgr.K01978;Eucgr.D01635;Eucgr.D01632
alpha-Linolenic acid metabolism	egr00592	2/17	28.5066667	0.08922507	0.00336734	Eucgr.C03551;Eucgr.C03543;Eucgr.C03541;Eucgr.C03214;Eucgr.H03323;Eucgr.H03327;Eucgr.H03340;Eucgr.H03343;Eucgr.H03330;Eucgr.H03342;Eucgr.H03338
Phenylpropanoid biosynthesis	egr00940	2/20	19.7592593	0.12349491	0.00643983	Eucgr.G01530;Eucgr.G01531;Eucgr.G01532;Eucgr.H00347;Eucgr.H00353;Eucgr.H00349;Eucgr.H00351
Stilbenoid, diarylheptanoid and gingerol biosynthesis	egr00945	1/5	201.875	0.00771843	0.00769938	Eucgr.G01530;Eucgr.G01531;Eucgr.G01532
Sesquiterpenoid and triterpenoid biosynthesis	egr00909	1/8	65.7959184	0.01975918	0.01961023	Eucgr.F04040;Eucgr.F04050;Eucgr.F04038;Eucgr.F04039;Eucgr.F04041
Other glycan degradation	egr00511	1/9	50.34375	0.02500772	0.02476182	Eucgr.J02456;Eucgr.J02454;Eucgr.J02455;Eucgr.J02459
ABC transporters	egr02010	1/9	50.34375	0.02500772	0.02476182	Eucgr.K00531;Eucgr.K00534
Cutin, suberine and wax biosynthesis	egr00073	1/10	39.7530864	0.03087373	0.0304901	Eucgr.H03009;Eucgr.H03002;Eucgr.H03013;Eucgr.H03011;Eucgr.H02999;Eucgr.H03005
Valine, leucine and isoleucine biosynthesis	egr00290	1/10	39.7530864	0.03087373	0.0304901	Eucgr.A00052;Eucgr.A00249;Eucgr.A00248
Photosynthesis - antenna proteins	egr00196	1/12	26.5785124	0.04445817	0.04363599	Eucgr.D00320;Eucgr.D00319;Eucgr.D00321;Eucgr.D00322;Eucgr.E02381

Column	Description
KO.ratio	Number of KEGG Orthology terms in geneset/number of KEGG Orthology terms annotated in the pathway)
Odds.ratio	The strength of association between geneset and annotated pathway
Expected	Expected number of terms in the geneset
P-value	P-value, Hypergeometric test

Supplemental Table 6- KEGG pathway enrichments for C.c.variegata specific gene family expansions.
Expansions are defined as orthogroups containing 5 or more genes and more than half of which are derived from C.c.variegata.

Pathway	PathwayID	KO.ratio	Odds ratio	Expected	Pvalue	GeneID
Phenylpropanoid biosynthesis	egr00940	2/17	30.5066667	0.08342956	0.00295293	Cocit.K0941;Cocit.K0940;Cocit.K0942;Cocit.K0938;Cocit.A0745;Cocit.A0695;Cocit.A0621;Cocit.A0622;Cocit.A0696;Cocit.A0629;Cocit.A0692;Cocit.K1629;Cocit.A0625; Cocit.A0624;Cocit.A0462;Cocit.A0743;Cocit.A0626;Cocit.A0627;Cocit.A0623;Cocit.A0630;Cocit.K1628;Cocit.A0698;Cocit.A0697;Cocit.K1630
Plant-pathogen interaction	egr04626	3/32	12.1391201	0.29561201	0.00296198	Cocit.H2724;Cocit.H2718;Cocit.H2729;Cocit.H2722;Cocit.H2719;Cocit.K1076;Cocit.K1086;Cocit.C1253;Cocit.C1485;Cocit.C1487;Cocit.K1142;Cocit.K1085
Ether lipid metabolism	egr00565	1/8	70.3877551	0.01847575	0.01834549	Cocit.C0826;Cocit.F0740;Cocit.C0827;Cocit.F0741;Cocit.C0828
Sesquiterpenoid and triterpenoid biosynthesis	egr00909	1/8	70.3877551	0.01847575	0.01834549	Cocit.C1575;Cocit.C1597;Cocit.C1739;Cocit.C1599
Cutin, suberine and wax biosynthesis	egr00073	1/10	42.5308642	0.02886836	0.02853282	Cocit.G0456;Cocit.G0663;Cocit.G0588;Cocit.G0587;Cocit.G0458

Column	Description
KO.ratio	Number of KEGG Orthology terms in geneset/number of KEGG Orthology terms annotated in the pathway)
Odds.ratio	The strength of association between geneset and annotated pathway
Expected	Expected number of terms in the geneset
P-value	P-value, Hypergeometric test

Supplementary Table 7- KEGG pathway enrichments for C.c. variegata specific gene family expansions that occurred after Eucalyptus and Corymbia diverged (Ks<=0.1).

KEGG Pathway Enrichments

Pathway	PathwayID	KO.ratio	Odds.ratio	Expected	Pvalue	GeneID
Sesquiterpenoid and triterpenoid biosynthesis	egr00909	1/8	70.3877551	0.01847575	0.01834549	Cocit.C1575;Cocit.C1597;Cocit.C1739;Cocit.C1599
Cutin, suberine and wax biosynthesis	egr00073	1/10	42.5308642	0.02886836	0.02853282	Cocit.G0456;Cocit.G0663;Cocit.G0588;Cocit.G0587;Cocit.G0458

Column	Description
KO.ratio	Number of KEGG Orthology terms in geneset/number of KEGG Orthology terms annotated in the pathway)
Odds.ratio	The strength of association between geneset and annotated pathway
Expected	Expected number of terms in the geneset
P-value	P-value, Hypergeometric test

Supplementary Table 8- KEGG pathway enrichments for E.grandis specific gene family expansions that occurred after Eucalyptus and Corymbia diverged (Ks<=0.1).

Pathway	PathwayID	KO.ratio	Odds.ratio	Expected	Pvalue	GeneID
Sesquiterpenoid and triterpenoid biosynthesis	egr00909	1/8	65.79591837	0.01975918	0.01961023	Eucgr.E00415;Eucgr.E00404;Eucgr.E00414;Eucgr.E00419
Other glycan degradation	egr00511	1/9	50.34375	0.02500772	0.02476182	Eucgr.J02456;Eucgr.J02459

Column	Description
KO.ratio	Number of KEGG Orthology terms in geneset/number of KEGG Orthology terms annotated in the pathway)
Odds.ratio	The strength of association between geneset and annotated pathway
Expected	Expected number of terms in the geneset
P-value	P-value, Hypergeometric test
