# Supplementary material for: Pests, diseases, and aridity have shaped the genome of Corymbia citriodora
Source: Commun Biol. 2021 May 10;4:537. doi: 10.1038/s42003-021-02009-0 (PMC8110574; doi:10.1038/s42003-021-02009-0)
Supplement: Supplementary file 2 — Description of Additional Supplementary Files [file 42003_2021_2009_MOESM2_ESM.pdf]

## Description of Additional Supplementary Files

**File name:** Supplementary Data File 1

**Description:** Formatted data for each of the genome tracks (A-F) in Figure 1. The data is formatted to be viewed using Circa software.

**File name:** Supplementary Data File 2

**Description:** The orthogroup data file for orthogroups among Corymbia, Eucalyptus, Vitis, Populus, and Salix. This data file was used to generate Figure 2a.

**File name:** Supplementary Data File 3

**Description:** Calculated pairwise synonymous mutation rates among single copy orthologs among Corymbia, Eucalyptus, Vitis, Populus, and Salix. This data file was used to generate Figure 2b.

**File name:** Supplementary Data File 4

**Description:** Pairwise synteny blocks among Corymbia and Eucalyptus. This data file was used to generate Figure 3.

**File name:** Supplementary Data File 5

**Description:** Pairwise synonymous mutation rates among paralogs within Corymbia, Eucalyptus and Vitis. This data was used to generate Figure 4a. Additionally, the line segments drawn between Corymbia paralogs stemming from each WGD event are included as well (Figure 4b). These line segments are formatted to be view using Circa software.

**File name:** Supplementary Data File 6

**Description:** CCV paralog pairs arising from Myrtales specific whole genome duplication event. Top protein hit for each paralog within Eucalyptus is provided.

**File name:** Supplementary Data File 7

**Description:** Genes maintained in syntenic blocks among Corymbia/Eucalyptus chromosome 3 and Populus chromosome 6 and 18.

**File name:** Supplementary Data File 8

**Description:** Pairwise synonymous mutation rates among: 1:1 orthologs between Corymbia and Eucalyptus; eucalypt expanded orthogroups; Corymbia specific orthogroup expansions and Eucalyptus specific expanded orthogroups.  Also includes a newick tree of the Corymbia terpene synthases.  Both of which were used to generate Figure 5.
