# Supplementary material for: Pests, diseases, and aridity have shaped the genome of Corymbia citriodora
Source: Commun Biol. 2021 May 10;4:537. doi: 10.1038/s42003-021-02009-0 (PMC8110574; doi:10.1038/s42003-021-02009-0)
Supplement: Supplementary file 11 — Reporting Summary [file 42003_2021_2009_MOESM11_ESM.pdf]

nature research

Corresponding author(s): Adam Healey
Last updated by author(s): Feb 19, 2021

Reporting Summary

Nature Research wishes to improve the reproducibility of the work that we publish. This form provides structure for consistency and transparency in reporting. For further information on Nature Research policies, see our [Editorial Policies](#) and the [Editorial Policy Checklist](#).

Statistics

For all statistical analyses, confirm that the following items are present in the figure legend, table legend, main text, or Methods section.

n/a	Confirmed	
☐	☒	The exact sample size (*n*) for each experimental group/condition, given as a discrete number and unit of measurement
☐	☒	A statement on whether measurements were taken from distinct samples or whether the same sample was measured repeatedly
☐	☒	The statistical test(s) used AND whether they are one- or two-sided<br>*Only common tests should be described solely by name; describe more complex techniques in the Methods section.*
☒	☐	A description of all covariates tested
☐	☒	A description of any assumptions or corrections, such as tests of normality and adjustment for multiple comparisons
☐	☒	A full description of the statistical parameters including central tendency (e.g. means) or other basic estimates (e.g. regression coefficient) AND variation (e.g. standard deviation) or associated estimates of uncertainty (e.g. confidence intervals)
☐	☒	For null hypothesis testing, the test statistic (e.g. *F*, *t*, *r*) with confidence intervals, effect sizes, degrees of freedom and *P* value noted<br>*Give P values as exact values whenever suitable.*
☒	☐	For Bayesian analysis, information on the choice of priors and Markov chain Monte Carlo settings
☒	☐	For hierarchical and complex designs, identification of the appropriate level for tests and full reporting of outcomes
☐	☒	Estimates of effect sizes (e.g. Cohen's *d*, Pearson's *r*), indicating how they were calculated

Our web collection on statistics for biologists contains articles on many of the points above.

Software and code

Policy information about availability of computer code

Data collection	As described in the Methods section. All open source or commercially available and previously published.
Data analysis	As described in the Methods section. All open source or commercially available and previously published.

For manuscripts utilizing custom algorithms or software that are central to the research but not yet described in published literature, software must be made available to editors and reviewers. We strongly encourage code deposition in a community repository (e.g. GitHub). See the Nature Research guidelines for submitting code & software for further information.

Data

Policy information about availability of data

All manuscripts must include a data availability statement. This statement should provide the following information, where applicable:

- Accession codes, unique identifiers, or web links for publicly available datasets
- A list of figures that have associated raw data
- A description of any restrictions on data availability

Sequencing data available in the SRA database under accessions: PRJNA234431,PRJNA333377, PRJNA333376, PRJNA333375, PRJNA333374. RNA sequencing data available at NCBI under BioProject PRJNA629009. Genome assembly deposited in DDBJ/ENA/GenBank under the accession JABURB000000000

Field-specific reporting

Ecological, evolutionary & environmental sciences study design

All studies must disclose on these points even when the disclosure is negative.

Study description	Generation of a reference genome sequence for Corymbia citriodora, and subsequent comparative analysis with Eucalyptus grandis to show expansions in ecologically important gene families.
Research sample	Corymbia citriodora subsp. variegata genotype CCV2-018 was selected for its wide use as a parent in the spotted gum breeding program of the Queensland Department of Agriculture and Fisheries, and its use for the generation of interspecific hybrids for investigating pulp and bioenergy production. Additionally, genotype CCV2-054 (genetic map parent) was collected from Woondum provenance around Gympie, Queensland and maintained as ramets.
Sampling strategy	Tissues for DNA and RNA extraction are as described in the manuscript.
Data collection	Data was collected as described in the Methods section.
Timing and spatial scale	Tissue samples were collected from ramets maintained at the glasshouse of the Queensland Department of Agriculture and Fisheries in Gympie, Queensland, Australia.
Data exclusions	No data excluded.
Reproducibility	RNASeq expression data was verified using a heat-map of normalized counts to ensure that expression among related tissues was consistent.
Randomization	Randomization is not needed for the analyses described in the manuscript.
Blinding	Blinding is not needed for the analyses described in the manuscript.

Did the study involve field work? ☐ Yes ☒ No

Reporting for specific materials, systems and methods

We require information from authors about some types of materials, experimental systems and methods used in many studies. Here, indicate whether each material, system or method listed is relevant to your study. If you are not sure if a list item applies to your research, read the appropriate section before selecting a response.

Materials & experimental systems

n/a	Involved in the study
☒	☐ Antibodies
☒	☐ Eukaryotic cell lines
☒	☐ Palaeontology and archaeology
☒	☐ Animals and other organisms
☒	☐ Human research participants
☒	☐ Clinical data
☒	☐ Dual use research of concern

Methods

n/a	Involved in the study
☒	☐ ChIP-seq
☒	☐ Flow cytometry
☒	☐ MRI-based neuroimaging
